# Selfish uptake versus extracellular arabinoxylan degradation in the primary degrader *Ruminiclostridium cellulolyticum*, a new string to its bow

**DOI:** 10.1186/s13068-022-02225-8

**Published:** 2022-11-19

**Authors:** Nian Liu, Séverine Gagnot, Yann Denis, Deborah Byrne, Craig Faulds, Henri-Pierre Fierobe, Stéphanie Perret

**Affiliations:** 1 Aix Marseille Univ, CNRS, LCB, Marseille, France, 31 chemin Joseph Aiguier F-13402, Marseille Cedex 20, Marseille, France; 2 Aix Marseille Univ, CNRS, IMM, Marseille, France; 3 INRAE, Aix Marseille Univ, INRAE, BBF, Marseille, France, 13009 Marseille, France

**Keywords:** ABC transporter, Solute binding protein, Arabinoxylan, Xylanase, AXOS

## Abstract

**Background:**

Primary degraders of polysaccharides play a key role in anaerobic biotopes, where plant cell wall accumulates, providing extracellular enzymes to release fermentable carbohydrates to fuel themselves and other non-degrader species. *Ruminiclostridium cellulolyticum* is a model primary degrader growing amongst others on arabinoxylan. It produces large multi-enzymatic complexes called cellulosomes, which efficiently deconstruct arabinoxylan into fermentable monosaccharides.

**Results:**

Complete extracellular arabinoxylan degradation was long thought to be required to fuel the bacterium during this plant cell wall deconstruction stage. We discovered and characterized a second system of “arabinoxylan” degradation in *R. cellulolyticum,* which challenged this paradigm. This “selfish” system is composed of an ABC transporter dedicated to the import of large and possibly acetylated arabinoxylodextrins, and a set of four glycoside hydrolases and two esterases. These enzymes show complementary action modes on arabinoxylo-dextrins. Two α-L-arabinofuranosidases target the diverse arabinosyl side chains, and two exo-xylanases target the xylo-oligosaccharides backbone either at the reducing or the non-reducing end. Together, with the help of two different esterases removing acetyl decorations, they achieve the depolymerization of arabinoxylo-dextrins in arabinose, xylose and xylobiose. The in vivo study showed that this new system is strongly beneficial for the fitness of the bacterium when grown on arabinoxylan, leading to the conclusion that a part of arabinoxylan degradation is achieved in the cytosol, even if monosaccharides are efficiently provided by the cellulosomes in the extracellular space. These results shed new light on the strategies used by anaerobic primary degrader bacteria to metabolize highly decorated arabinoxylan in competitive environments.

**Conclusion:**

The primary degrader model *Ruminiclostridium cellulolyticum* has developed a “selfish” strategy consisting of importing into the bacterium, large arabinoxylan–dextrin fractions released from a partial extracellular deconstruction of arabinoxylan, thus complementing its efficient extracellular arabinoxylan degradation system. Genetic studies suggest that this system is important to support fitness and survival in a competitive biotope. These results provide a better understanding of arabinoxylan catabolism in the primary degrader, with biotechnological application for synthetic microbial community engineering for the production of commodity chemicals from lignocellulosic biomass.

**Supplementary Information:**

The online version contains supplementary material available at 10.1186/s13068-022-02225-8.

## Background

Lignocellulosic biomass represents an unlimited renewable carbon source on Earth. It consists mainly of plant cell wall polysaccharides, of which arabinoxylan is one of the main ones. Its fermentation contributes to global carbon recycling and is of interest for the conversion of plant biomass into biofuel or other valuable chemicals [1]. Anaerobic communities play a key role in the fermentation process of plant cell wall polysaccharides. They are found in various biotopes where decomposing plants accumulates, such as in anaerobic digesters, soils, sewage sludges, organic wastes, animal manure, and in the rumen and gastrointestinal tracts of monogastric animals… These biotopes host complex consortia of metabolically diverse micro-organisms, such as hydrolytic, acid forming, acetogenic, and methanogenic bacteria that interact together. The primordial step for the development of these communities is the hydrolysis of plant cell wall polysaccharides, which is carried out by a few primary degraders making fermentable sugars available for other bacteria [2]. Recently, investigations on the use of microbial consortia rather than pure culture to produce chemicals of interest from lignocellulosic biomass have gained growing interest [3]. In this context, a better understanding of the metabolic strategies used by a primary degrader living in complex environments is desired.

The plant cell wall is mainly composed of cellulose and hemicellulose, xylan is the most ubiquitous and prominent constituent of hemicellulose. Xylan forms a heterogeneous group of branched polysaccharides in which the backbone forms a long chain of β-1,4 linked D-xylopyranosyl units. In arabinoxylan, xylose residues can be mono- or disubstituted with the α-L-arabinofuranosyl unit (α-1,2 or α-1,3 linkage), acetylated, and arabinose residues can be esterified with aromatic residues, such as ferulic or *p*-coumaric acid [4]. Due to its heterogeneous structure, the depolymerization of xylan requires the action of several carbohydrate-active enzymes (CAZymes), glycoside hydrolases (GH), such as endoxylanases, and β-xylosidases which target the main chain of xylan and α-arabinofuranosidases which hydrolyze the arabinosyl side chains, and carbohydrate esterases (CE) that target the esterified units [5, 6]. Complete extracellular depolymerization of xylan is not necessary for the bacteria to grow on this substrate. For instance, some bacteria found in the human colon, organic wastes, or soils, developed original systems to import and depolymerize intracellular sugars, such as xylo-oligosaccharides (XOS), arabinoxylo-oligosaccharides (AXOS), degradation products from glucuronoxylan, as well as arabinan or galactans [7–17]. In these bacteria, ABC transporters carry out the import process. They comprise an extracellular membrane-bound Family 1-Solute Binding Protein (SBP), which captures and delivers oligosaccharides to its corresponding channel formed by two membrane proteins (transmembrane domain, TMD). The import process is driven by ATP hydrolysis achieved by two proteins called Nucleotide Binding Domain (NBD) [18]. Some of these bacteria produce some extracellular endoxylanases that cleave the arabinoxylan backbone and release a mixture of XOS and AXOS [7, 8, 10, 12, 17]. Others, which do not secrete xylanase, are unable to grow on arabinoxylan but, by cross-feeding use fermentable dextrins released by xylanolytic organisms called primary degraders [9, 11, 19].

*Ruminiclostridium cellulolyticum* is an anaerobic bacterium of interest because of its ability to degrade and grow on a diversity of polysaccharides, such as cellulose, or hemicellulose, such as xylan or xyloglucan [20–22]. Like other (hemi)-cellulolytic bacteria, it plays a crucial role in anaerobic environmental biotopes [23, 24]. This Gram-positive bacterium is an excellent model to study the anaerobic degradation of plant cell wall polysaccharides, as it is one of the few for which genetic tools have been developed to inactivate or overexpress genes, allowing in vivo experiments to be designed [21, 25–31]. Its hydrolytic skills are primarily related to its ability to produce highly active extracellular enzymatic complexes called cellulosomes. These complexes efficiently depolymerize xylan chains, releasing mainly arabinose, xylose, xylobiose, and xylotriose [20, 32]. Furthermore, as the strain grows quite fast when the monosaccharides xylose or arabinose are used as the unique carbon source, it was long believed that *R. cellulolyticum* only imports elemental monosaccharides generated from arabinoxylan.

In the present work, we describe a new gene cluster termed *xua* (xylan utilization associated), encoding an ABC transporter and cytoplasmic xylan degradation enzymes that import and depolymerize arabinoxylan dextrins in *R. cellulolyticum*, respectively. This raises the question of the utility of such an intracellular degradation facility in the metabolic strategy of the strain, since it has an efficient extracellular cellulosomal arabinoxylan depolymerization system. This intracellular system of degradation involves a set of four GHs attacking complex arabinoxylo-dextrins with complementary modes of action and two acetyl esterases. We show for the first time, direct genetic evidence that it strongly enhances the fitness of this primary degrader during growth on arabinoxylan, revealing that a substantial part of arabinoxylan hydrolysis is carried out in the bacterial cytosol. These results shed new light on the understanding of arabinoxylan degradation strategy by primary biocatalysts that could be advantageous when evolving in anaerobic microbial communities.

## Results

### Sequence analysis of xua gene cluster and its products

**Fig. 1:**
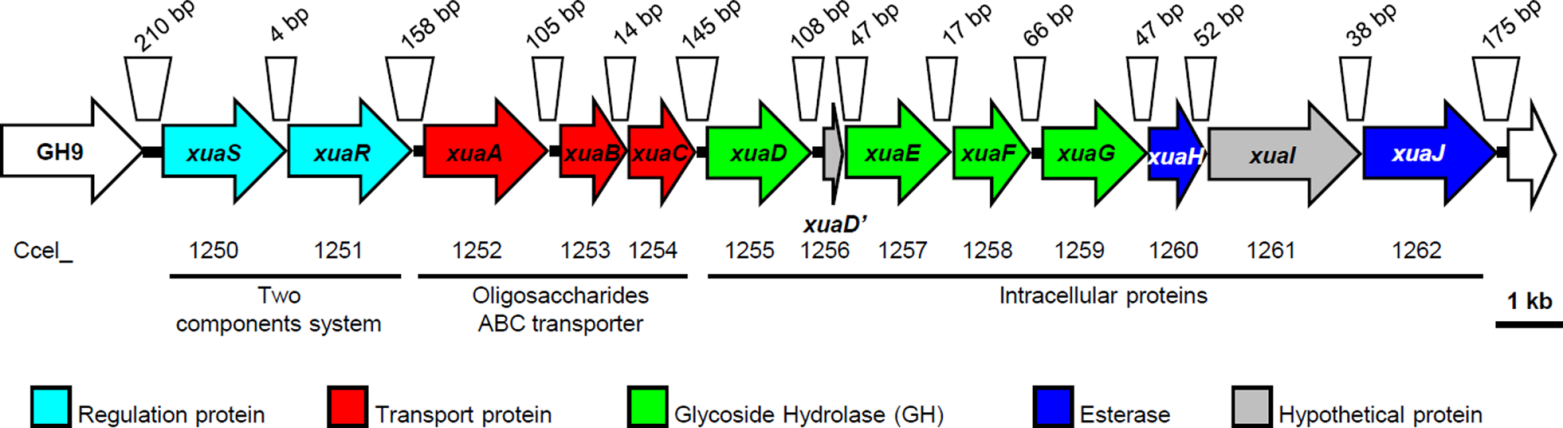
*xua* gene cluster and its neighboring genes The size of intergenic regions is indicated above the genes, and the locus number of the genes and predicted function(s) of the encoded proteins are indicated below the genes according to the color code

The x*ua* gene-cluster encompasses 13 genes occupying loci Ccel_1250 to Ccel_1262 (Fig. 1). These genes encode a putative two-component system composed of a sensor (XuaS) and a response regulator belonging to the AraC family (XuaR); a putative solute binding protein named XuaA and two integral membrane proteins XuaB and C forming together a putative ABC-transporter; and a set of four predicted glycoside hydrolases (XuaD, E, F, G), two putative esterases (XuaH and J), and two proteins of unknown function(s) (XuaD’ and XuaI).

Upstream and downstream of the cluster, the genes at loci Ccel_1249 and Ccel_1263, respectively, encode a secreted cellulosomal GH9 cellulase (Cel9T) [33], and a putative regulator protein of the FadR family. Intergenic regions in the cluster are short, between 4 and 158 bp-long, suggesting an operon-like organization (Fig. 1).

XuaA is predicted to be a Family-1 ABC transporter solute binding protein (SBP). This family includes proteins mainly binding to oligosaccharides [34, 35]. XuaA has a typical Gram-positive lipoprotein signal peptide at its N-terminus, indicating that the protein might be exported and membrane-anchored. XuaB and XuaC are putative transmembrane domains predicted to contain six transmembrane-spanning helices. Their sequences contain the consensus sequence [EAA-X3-G-X9-I-X-LP] typically found in integral membrane proteins of ABC-transporters [18, 36].

The genes *xuaD*, *E*, *F,* and *G* encode putative different GHs belonging to different families. No signal peptide is predicted for any of these proteins, suggesting localization in the cytosol. XuaD belongs to the GH51 family that comprises endoglucanases, endo-β-1,4-xylanases, β-xylosidases, α-L-arabinofuranosidases, or cellobiohydrolases. XuaE belongs to the GH43 family, subfamily 10, which includes enzymes with xylan β-xylosidase or α-L-arabinofuranosidase activities. XuaF belongs to the GH8 family, in which enzymes with diverse substrate specificities are found (cellulases, chitosanase, licheninase, xylanase). XuaG is classified in the GH39 family that includes β-xylosidase α-L-arabinofuranosidase, β-glucosidase, β-galactosidase, α-L-iduronidase (CAZy). The products of the genes *xuaH*, and *xuaJ* are annotated as putative esterase and putative acetyl esterase, respectively, in the NCBI database. Both enzymes also lack a signal peptide sequence. Finally, the genes *xuaD’* and *xuaI* encode proteins of unknown function(s) of 88 and 732 amino acids, respectively, also lacking a signal peptide sequence.

### Genes of the *xua* cluster are regulated by arabinoxylan

**Fig. 2:**
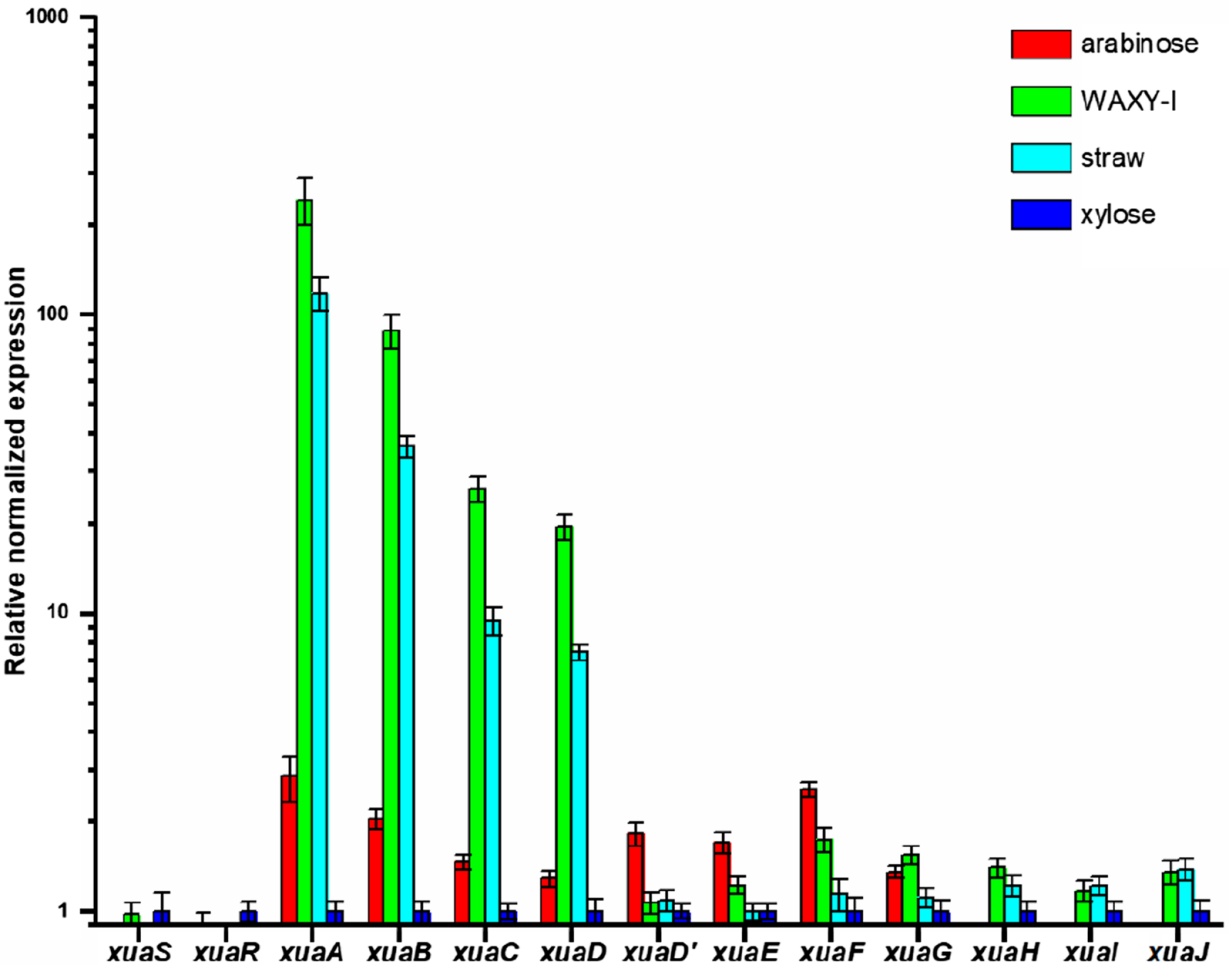
qPCR analysis of mRNA produced by *R. cellulolyticum* Total RNA was extracted from cultures of *R. cellulolyticum* grown in minimal medium supplemented with 2 g L^−1^ arabinose, 2 g L^−1^ WAXY-I (arabinoxylan), 5 g L^−1^ wheat straw or 2 g L^−1^ xylose as the sole carbon source. Normalization was performed using the 16S RNA encoding-gene and gene expression level is given relative to xylose culture. Error bars indicate the standard deviations of three independent experiments

The predicted functions of the encoded proteins suggest the cluster is involved in arabinoxylan utilization. We, therefore, tested the expression level of these genes on growth substrates containing arabinoxylan polysaccharides or on monosaccharides found in arabinoxylan (Fig. 2). A basal expression level of the genes *xuaS* and *xuaR* was observed on all carbon sources tested. In contrast, the expression of the genes *xuaA* to *xuaD* encoding the ABC-transporter components and the first enzyme of the cluster is induced 7–240-fold when the cells are grown on arabinoxylan or wheat straw. These genes are primarily expressed in the presence of arabinoxylan, rather than monosaccharides constituting the arabinoxylan. Interestingly, the expression level of the genes decreases as the distance to *xuaA* increases, suggesting an operon structure encompassing at least *xuaA* to *xuaD*. The expression of the genes located downstream *xuaD* does not seem to be induced in our conditions.

### XuaA targets arabinoxylan dextrins

**Fig. 3:**
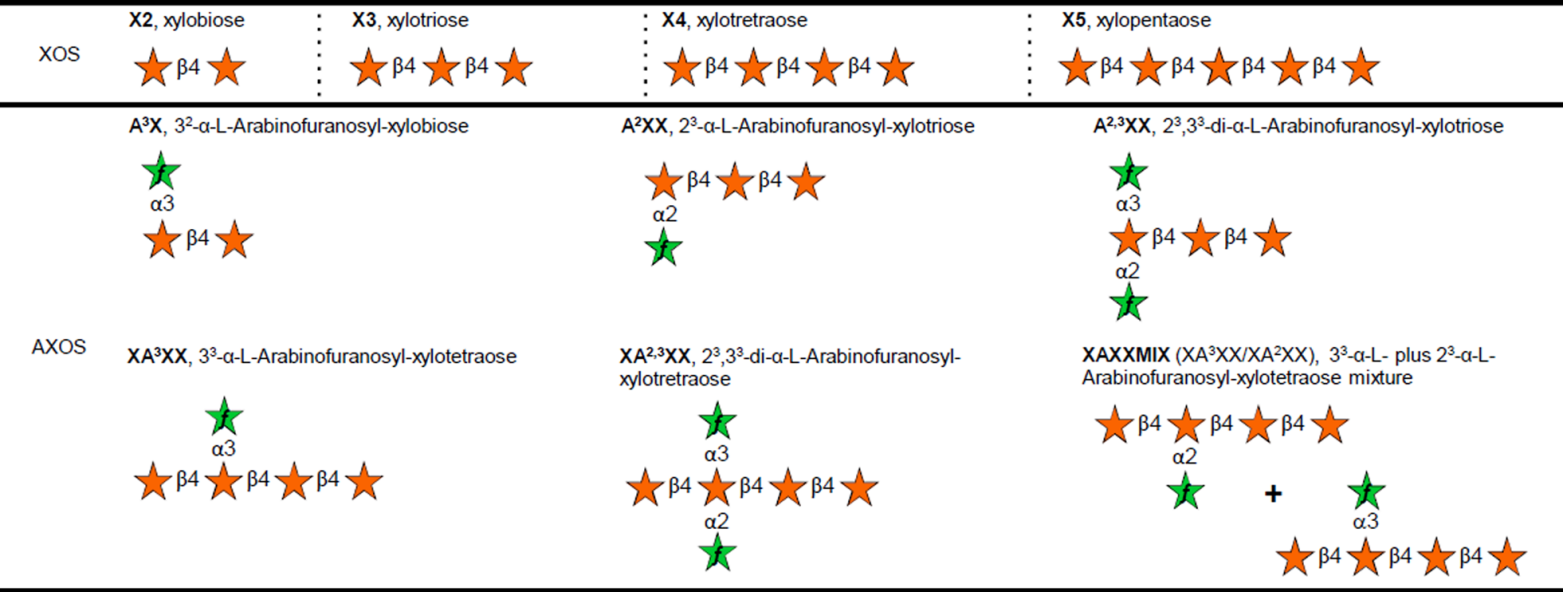
Schematic representation of xylo-oligosaccharides and arabinoxylo-oligosaccharides used in this study Stars represent xylopyranose (orange) and arabinofuranose (green)

The binding of XuaA to the various ligands was first screened using TSA, and the binding parameters were subsequently determined by ITC (Table 1, Fig. 3, Additional files 1 and 2). XuaA does not bind to the monosaccharide arabinose, xylose, and glucose nor to the unbranched XOS, but it binds to decorated arabinoxylan dextrins containing two to four xyloses and carrying one or two α-1,2 or α-1,3 arabinosyl side chains. The *K*_*D*_ ranged between 12 to 63 nM depending on the tested sugar (Table 1). The affinity was found stronger for arabinoxylan dextrins displaying xylose residue bearing two arabinosyl side chains. The affinity is decreased for α-1,3 arabinose substitution or shorter main chain backbone. The measured Δ*G* were similar for all interacting sugars. In all cases, the interactions are characterized by a strong enthalpic contribution with an unfavorable entropic contribution.

**Table 1:** Binding of XuaA to AXOS and XOS

Ligand	TSA	ITC				
	∆T (°C)	N (sites)	*K*_D_ (nM)	∆*G*	∆*H*	-T∆*S*
				(kcal mol^−1^)	(kcal mol^−1^)	(kcal mol^−1^)
A^2,3^XX	13.5	0.796 ± 2.37e-2	12.0 ± 4.6	− 10.8 ± 0.3	− 26.0 ± 2.6	15.1 ± 2.8
XA^2,3^XX	14	1.05 ± 5.67e-2	15.9 ± 0.1	− 10.6 ± 0.0	− 15.5 ± 0.5	4.8 ± 0.5
XA^3^XX	11.0	0.652 ± 2.83e-3	40.4 ± 9.3	− 10.1 ± 0.1	− 21.7 ± 1.1	11.6 ± 1.2
A^3^X	9.0	1.06 ± 7.97e-2	63.1 ± 16.2	− 9.8 ± 0.2	− 18.8 ± 0.3	9.0 ± 0.4
A^2^XX	6.5	NB				
X4	0	NB				
X2, X3, X5, X6	0	ND				
A, X, G	0	ND				

TSA and ITC values are the mean of at least 2 experiments. Xylohexaose (X6); Arabinose (A); xylose (X); glucose (G); other sugars refer to Fig. 3, TSA and ITC data are presented in Additional file 1 and 2, respectively

No binding *NB*, not determined *ND*

### XuaD and XuaE are α-L-arabinofuranosidases

XuaD, classified as a GH51, cleaves α-1,2 or α-1,3 linked arabinosyl residues from mono-substituted xylosyl residues like those found in A^3^X, A^2^XX, XA^3^XX, or XA^2^XX (Table 2). In presence of doubly substituted AXOS (A^2,3^XX and XA^2,3^XX), the enzyme can only remove both arabinosyl decorations when the double-decorated xylosyl residue is located at the non-reducing extremity of the AXOS (A^2,3^XX) (Additional file 3). This enzyme displays similar *K*_m_ and *k*_cat_ values with AXOS carrying a single arabinosyl decoration or a double-substitution at the non-reducing end extremity (Table 2, Additional file 5).

**Table 2:** Catalytic parameters of XuaD, E, F, G, H and J

		XuaD	XuaE	XuaF	XuaG	XuaH	XuaJ
AXOS	A^3^X	IU: 2381.7 ± 6.7	NA	NA	NA	ND	ND
		Products: Ara, X2					
	A^2^XX	IU: 1263.0 ± 46.1	NA	IU: 202.3 ± 18.7	NA		
		*K*_m_ 1.4 ± 0.3		Products: Xyl, A^2^X?			
		*k*_cat_ 3408.0 ± 356.6					
		*k*_cat_/*K*_m_ 2411.9 ± 303.3					
		Products: Ara, X3					
	A^2,3^XX	IU: 1256.3 ± 50.2	IU: 1938.0 ± 109.6	ND	NA		
		*K*_m_ 1.8 ± 0.2	*K*_m_ 0.1 ± 0.003				
		*k*_cat_ 4191.3 ± 117.3	*k*_cat_ 2253.5 ± 142.1				
		*k*_cat_/*K*_m_ 2388.2 ± 182.9	*k*_cat_/*K*_m_ 23137.8 ± 1355.0				
		Products: Ara, X3	Products: Ara, A^2^XX, A^3^XX?				
	XA^3^XX	IU: 1218.9 ± 38.5	IU: 1.6 ± 0.05	IU: 166.7 ± 12.8	IU: 21.2 ± 0.2		
		Products: Ara, X4	Products: Ara, X4	Products: Xyl, XA^3^X ?	Products: Xyl, A^3^XX?^a^		
	XAXXMIX	IU: 1325.8 ± 7.8	NA	ND	IU: 18.0 ± 0.3		
		Products: Ara, X4			Products: Xyl, A^2^XX, A^3^XX ?^a^		
	XA^2,3^XX	IU: 1.5 ± 0.03	IU: 1927.2 ± 100.4	IU: 29.7 ± 2.8	IU: 20.3 ± 0.2	NA	NA
		Products: Ara, X4	Products: Ara, XA^2^XX, XA^3^XX	Products: Xyl, XA^2,3^X ?	Products: Xyl, A^2,3^XX^a^		
XOS	X5	ND	ND	IU: 25.3 ± 0.7	IU: 6.2 ± 0.5	ND	ND
				Products: Xyl, X4, X3	Products: Xyl, X4, X3, X2^a^		
	X4	ND	ND	IU: 87.2 ± 7.6	IU: 23.3 ± 0.5		
				Products: Xyl, X3, X2	Products: Xyl, X3, X2^a^		
	X3	ND	ND	IU: 158.7 ± 21.1	IU: 24.5 ± 1.6		
				Products: Xyl, X2	Products: Xyl, X2^a^		
	X2	ND	ND	NA	NA		
1-Naphthyl acetate		ND	ND	ND	ND	IU: 3504.6 ± 18.9	IU: 21130.4 ± 676.4
						*K*_m_: 3.5 ± 1.1	*K*_m_: 0.2 ± 0.02
						*k*_cat_: 5435.7 ± 846.4	*k*_cat_: 21380.0 ± 440.0
						*k*_cat_/*K*_m_: 1628.1 ± 295.7	*k*_cat_/*K*_m_: 88703.9 ± 7849.6

^a^Transglycosylation was detected

Specific activity (IU) is given in IU/µmol of enzyme; When measured, *K*_m_ is given in mM, *k*_cat_ in min^−1^, and *k*_cat_/*K*_m_ in min^−1^ mM^−1^

*NA* not active, *ND* not determined

**Fig. 4:**
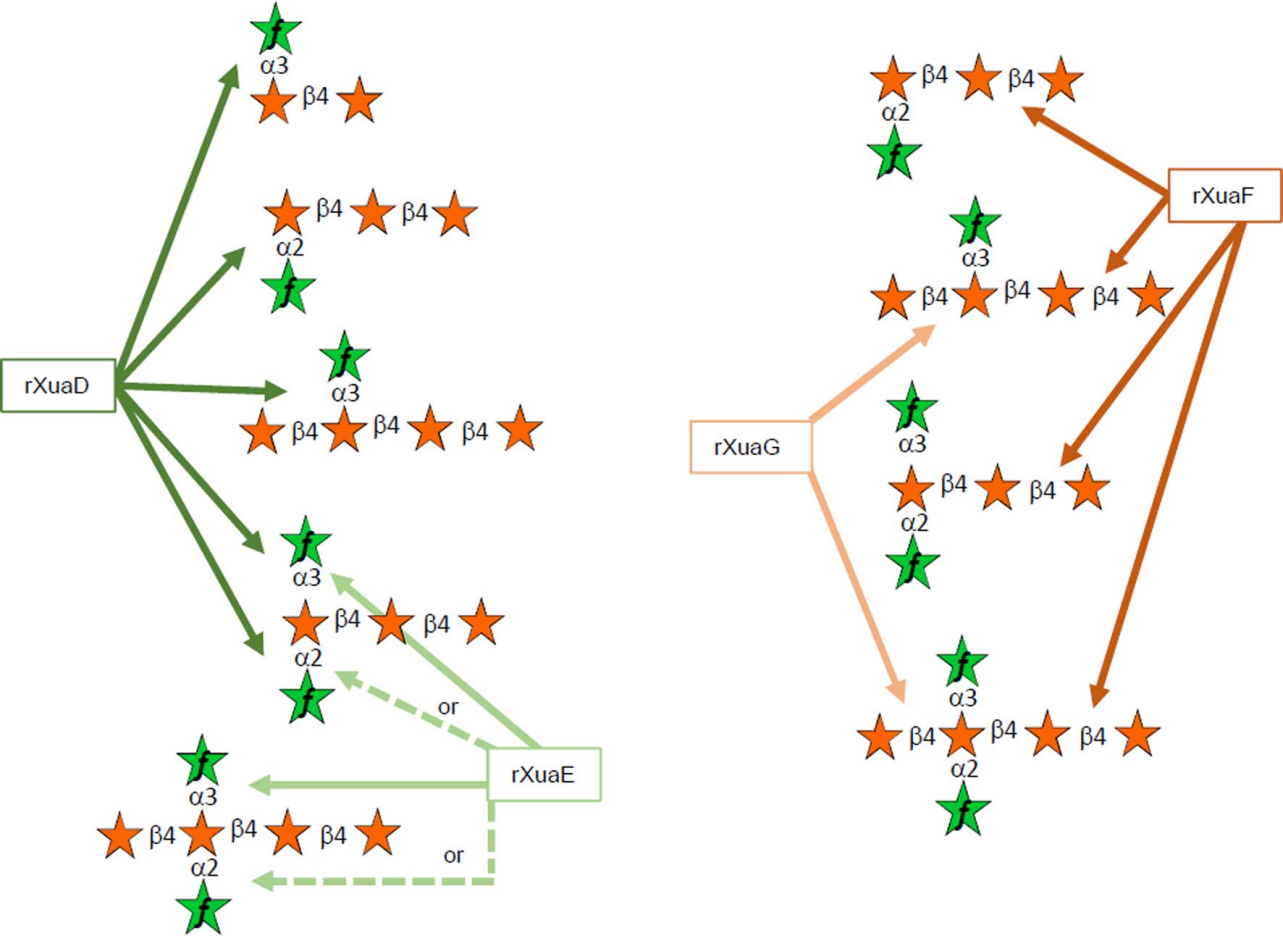
Specificity of XuaD, E, F and G for AXOS Arrows represent the glycosidic linkage targeted by the Xua enzymes. The dotted line represents alternative cleavage for XuaE which cleaves either the α-1,2 or the α-1,3 arabinosyl residues on double-substituted xylosyl residues located at the non-reducing end or in the middle of the AXOS

XuaE, classified as a GH43_10, cleaves either the α-1,2 or the α-1,3 arabinosyl residues on double-substituted xylosyl residues located at the non-reducing end or in the middle of the AXOS (A^2,3^XX and XA^2,3^XX, respectively) (Table 2, Additional file 3 and 5). In contrast to XuaD, XuaE is not active toward mono-substituted AXOS, regardless of the type of glycosidic link (α-1,2 or α-1,3) or the position of the substituted xylosyl residue within the AXOS (A^2^XX, A^3^X, XA^3^XX or the mix of XA^2^XX and XA^3^XX). Determination of the catalytic parameters of XuaE on the disubstituted A^2,3^XX revealed an 18-fold lower *K*_m_ than found for XuaD, and a twofold lower *k*_cat_ value than for XuaD (Table 2, Additional file 5). These data indicate that both enzymes target arabinosyl decorations but with different and complementary specificities. Together they can remove all types of arabinosyl substitutions from imported AXOS and generate arabinose-free xylodextrins in the cytosol (Fig. 4).

### XuaF and XuaG are exo-xylanases

XuaF is classified as a member of the GH8 family. This enzyme is active on both AXOS and XOS, releasing at least xylose thereby indicating that XuaF is a β-xylosidase. In addition to xylose, the enzyme releases other types of dextrins from AXOS that could not be identified by HPAEC–PAD as the corresponding oligosaccharides are not commercially available. However, on XA^2,3^XX the enzyme releases xylose and an AXOS for which the corresponding peak is probably superimposed to the peak of the substrate and could correspond to XA^2,3^X (Additional file 4). With this substrate, XuaF, however, did not generate A^2,3^XX, it can, therefore, be deduced that XuaF likely hydrolyzes XA^2,3^XX at the reducing end and is, therefore, a reducing-end-xylose releasing exo-oligoxylanase. Compared with X4, the presence of a double-substituted xylose residue in XA^2,3^XX reduces the specific activity of the enzyme. Nevertheless, XuaF displays a higher activity on monosubstituted XA^3^XX compared with that of X4, thus showing that a single arabinosyl decoration boosts its activity, whereas double-arabinosyl substitutions are damaging (Table 2). XuaF releases xylose and short xylodextrins and is not active on X2. Specific activities are inversely proportional to the size of the XOS.

XuaG is annotated within the GH39 family. Xylose is released after incubation of the enzyme with AXOS harboring an undecorated xylosyl residue at the non-reducing end and XOS (Table 2, Additional file 4). Arabinose decoration(s) present on the xylose located at the non-reducing end, therefore, blocks the activity of the enzyme (A^3^X, A^2^XX, A^2,3^XX) (Table 2). When incubated with XA^2,3^XX, XuaG releases xylose and A^2,3^XX in contrary to XuaF (Additional file 4). Our data, therefore, show that XuaG is a non-reducing end exo-oligoxylanase and that removal of arabinose decoration(s) at the non-reducing end of the dextrin is crucial for its action.

In summary, XuaF and XuaG remove xylose from the reducing end and the non-reducing end of the main chain, respectively (Fig. 4). The activity of XuaG is hampered by arabinosyl decoration on the xylosyl residue at the non-reducing extremity. However, as mentioned above, arabinosyl decorations can be efficiently removed by the arabinofuranosidases XuaD and XuaE, which target mono-substituted (XuaD), double-substituted xylosyl residues at the non-reducing end (XuaD or XuaE) or occupying a central position of the AXOS (XuaE, then XuaD). Thus, all tested AXOS can be ultimately converted by the four above-mentioned enzymes into arabinose, xylose, and xylobiose (Fig. 4).

### XuaH and XuaJ are esterases

XuaH and J are annotated as putative esterase in NCBI. XuaH belongs to the family A85-esteraseD-FGH in the ESTHER database. This protein shares 55% identity with an acetylesterase from *Caldicellosiruptor saccharolyticus* (XynC) encoded in a cluster of genes involved in xylan degradation [37]. XuaJ has no homologous proteins in the ESTHER database but is classified in the newly created CE20 family in the CAZy database. In this family the only characterized protein XacXaeA (XAC1771) from *Xanthomonas citri* pv. citri str. 306, is a xyloglucan acetyl esterase [38]. XuaJ shares 36.8% identity with this protein.

Activity studies using 1-naphthyl acetate as the substrate indicate that both XuaH and J have acetyl esterase activity but with different *K*_m_ and *k*_cat_ (Table 2, Additional file 5). These enzymes might be involved in the removal of acetyl decorations found in the imported AXOS, putatively targeting different positions of the acetyl on arabinoxylan [39]. This result suggests that all acetyl decorations do not have to be extracellularly removed before the uptake of the oligosaccharides by *R. cellulolyticum*.

### XuaA increases the fitness of the bacteria during growth on arabinoxylan

**Fig. 5:**
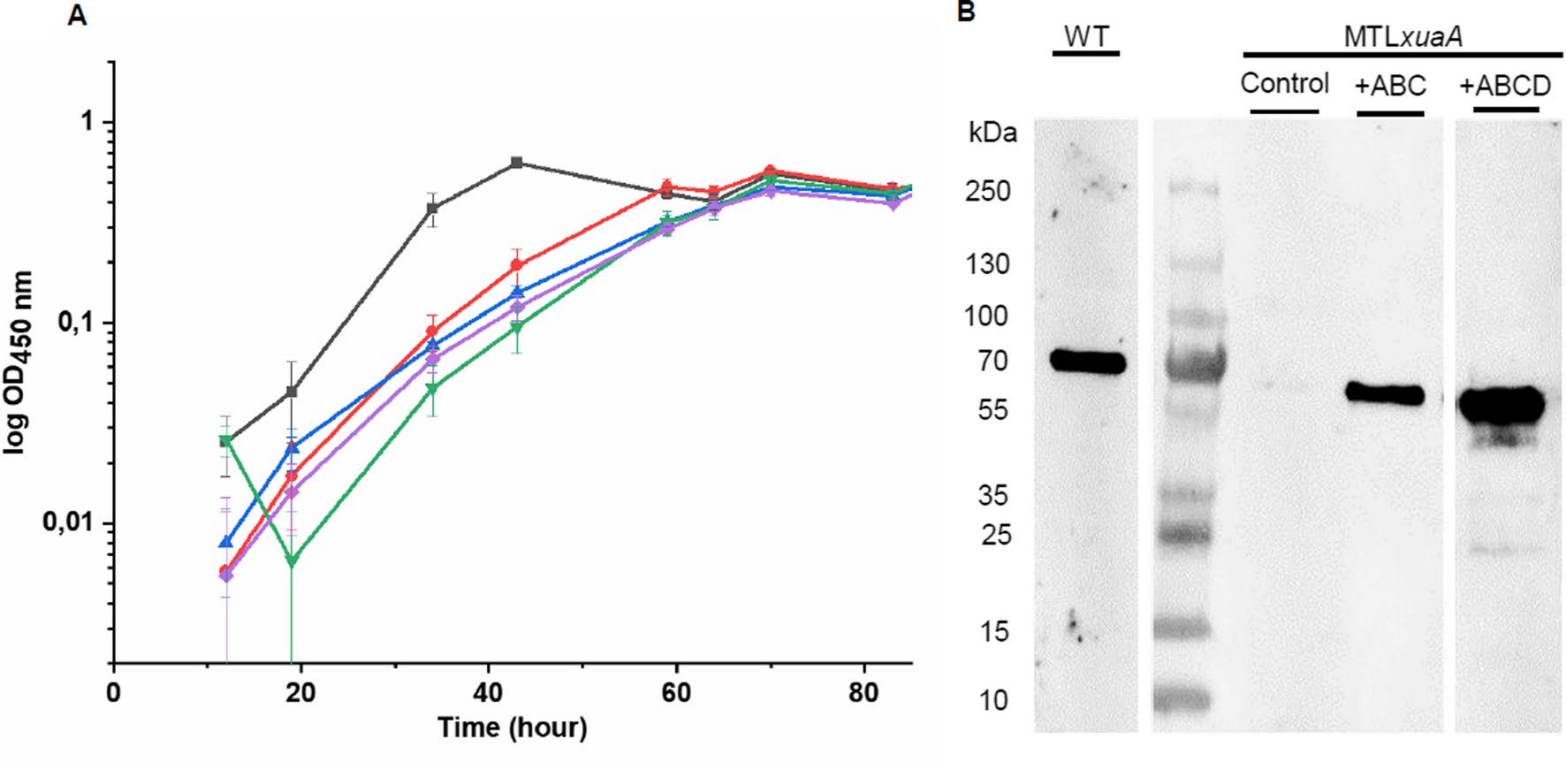
Characterization of *R. cellulolyticum* wild-type and MTL*xuaA* derivative strains A. Growth curve of different strains grown in minimal medium containing 2 g L^−1^ WAXY-I. The strains are: WT strain (gray), MTL*xuaA* strain (red), MTL*xuaA* strain carrying an empty vector (blue), MTL*xuaA* strain carrying pSOS*xuaABC* (green), MTL*xuaA* strain carrying pSOS*xuaABCD* (purple). Experiments were performed in triplicate and bars indicate the standard deviations. B. Western blot analysis of cells obtained from *R. cellulolyticum* wild-type, MTL*xuaA* containing the vectors pSOSzeroTm (Control), pSOS*xuaABC* (+ ABC) or pSOS*xuaABCD* (+ ABCD)

*R. cellulolyticum* produces cellulosomes in the extracellular environment, which are a highly efficient enzymatic complex to degrade arabinoxylan [20, 27, 32]. The presence of a second cluster dedicated to arabinoxylan utilization encoding cytosolic enzymes and an ABC-transporter that uptakes large AXOS for their subsequent depolymerization prompted us to examine the usefulness of this new system of intracellular “arabinoxylan” utilization in the physiology of this true primary plant cell wall degrader. To analyze the in vivo function of the new intracellular system, we inactivated the gene *xuaA* by insertion of a type II intron using the ClosTron tool to disable AXOS uptake (Additional file 6). Western blot confirmed the absence of XuaA in the mutant MTL*xuaA* strain in cell extracts (Fig. 5). The growth of the mutant strain in a minimal medium containing arabinose as the sole carbon source is similar to that of the wild-type strain, indicating that inactivation has no effect on monosaccharide utilization by the mutant strain (Additional file 7). When using WAXY-I as the sole carbon source, the growth of the mutant is characterized by a twofold increased doubling time, compared to the wild-type strain. The end of the exponential phase of growth is thus delayed by around 20 h compared with the wild-type strain thus emphasizing the importance of AXOS import and intracellular degradation system during the growth on this branched polysaccharide (Fig. 5). Complementation studies were attempted by introducing into the mutant strain a vector expressing either the genes *xuaABC* (pSOS*xuaABC*), or the genes *xuaABCD* (pSOS*xuaABCD*), or an “empty” vector (pSOSzeroTm). The genes *xuaABCD* were chosen because of their higher expression level on cultures containing arabinoxylan in the growth substrate (WAXY-I or wheat straw) (Fig. 2). Western blot analysis confirmed the synthesis of XuaA in the wild-type strain and in the mutant strains hosting the vectors pSOS*xuaABC* or pSOS*xuaABCD*. When grown on WAXY-I, the doubling-time of the mutant strain MTL*xuaA*(pSOS*xuaABC*) and MTL*xuaA*(pSOS*xuaABCD*) were similar to that determined for the mutant strain MTL*xuaA* and MTL*xuaA*(pSOSzeroTm). Thus, the introduction of the genes *xuaABC* or *xuaABCD* in *trans* in the mutant MTL*xuaA* failed to restore a wild-type phenotype (Fig. 5). This result can be explained by the fact that the intron inserted in *xuaA* might induce a polar effect on the expression of the downstream genes as already observed for other ClosTron interrupted gene clusters [28, 29]. As a consequence, the *xua* cluster might form a transcriptional unit starting from *xuaA* and going beyond *xuaD*, suggesting that the downstream genes whose expression is impaired in the mutant strains are critical for the fitness of the bacterium when using arabinoxylan as the carbon source despite of their weak expression level.

### Transcriptional organization of *xua* genes

**Fig. 6:**
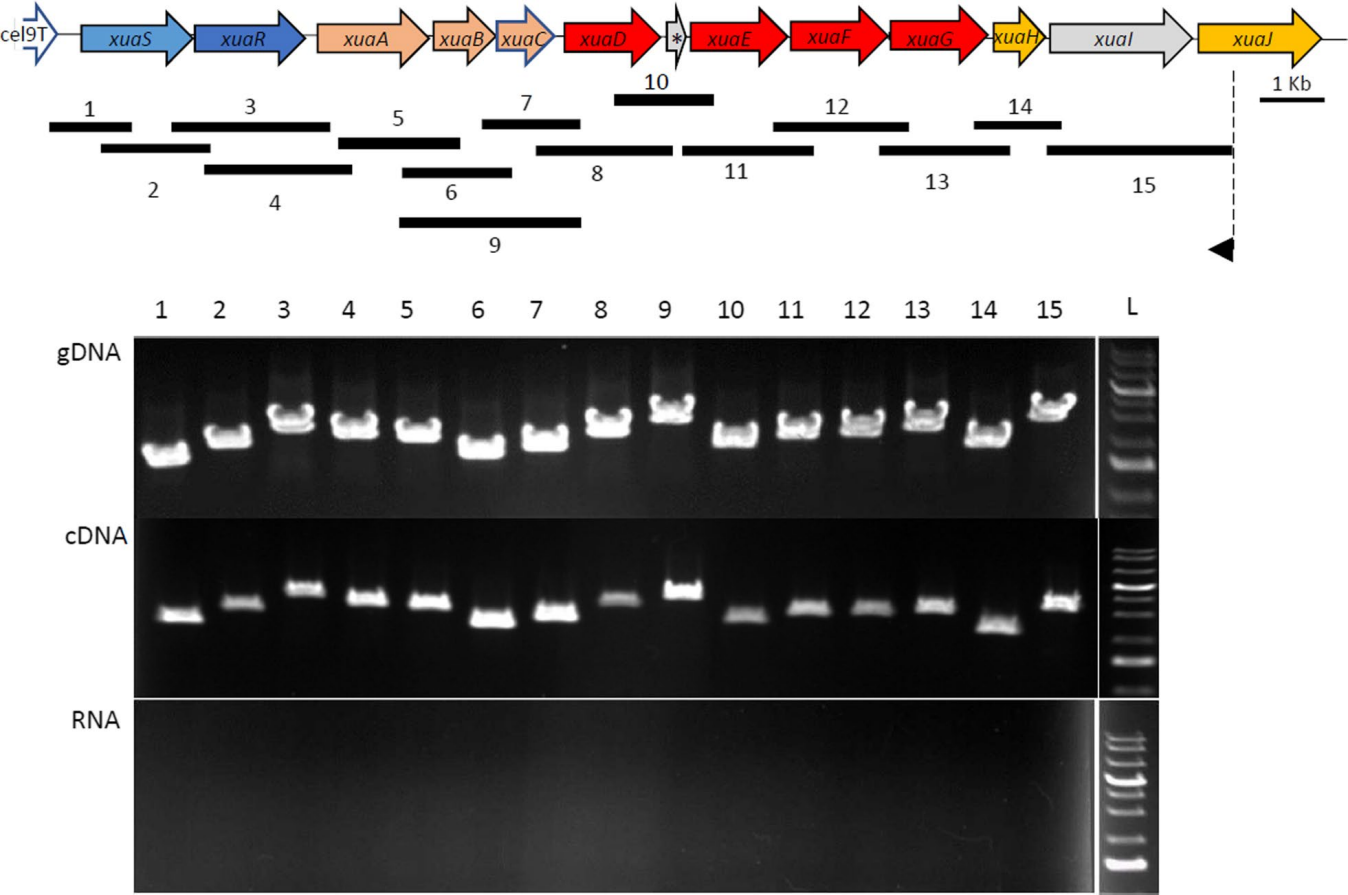
Transcriptional intergenic links in the *xua* cluster The *xua* cluster is shown at the top (star corresponds to *xuaD’*). Just below, the black lines correspond to the PCR amplifications. PCR amplifications were performed on genomic DNA (gDNA, upper electrophoresis gel), cDNA (middle electrophoresis gel) obtained after reverse transcription from an arabinoxylan (WAXY-I) grown culture, or mRNA (lower electrophoresis gel). The primers used to study the transcriptional links between two successive genes were localized at the end of the first gene for the direct primer and at the beginning of the second gene for the reverse primer. The amplicons were obtained using the following primer pairs: 1 (1249-E-RT-up/qRT-*xuaS*-708-rev); 2 (qPCR-*xuaS*-431-dir/1251-S-RT-do); 3 (1250-E-RT-up and 1252-S-RT-do); 4 (qPCR-*xuaR*-277-dir/qRT-*xuaA*-525-rev); 5 (qPCR-*xuaA*-2nd-378-dir/qPCR-*xuaB*-584-rev); 6 (1252-E-RT-up-2nd/1254-S-RT-do-3rd); 7 (1253-E-RT-up/1255-S-RT-do); 8 (1254-E-RT-up/1256-S-RT-do); 9 (1252-E-RT-up-2nd/1255-S-RT-do); 10 (1255-E-RT-up-2nd/1257-S-RT-do); 11 (1256-E-RT-up/1258-S-RT-do); 12 (1257-E-RT-up/1259-S-RT-do); 13 (1258-E-RT-up/1260-S-RT-do); 14 (1259-E-RT-up/1261-S-RT-do); 15 (1260-E-RT-up/1262-S-RT-do)

To confirm the organization of the cluster as an operon encompassing the *xua* genes, transcriptional links between two and/or three successive *xua* genes from *xuaS* to *xuaJ* were analyzed by RT-PCR. The cDNA was synthesized from a specific primer hybridizing to *xuaJ*. All the PCR tested with cDNA as the matrix resulted in amplicons of the same size as the PCR performed on gDNA, and no amplifications were obtained with the control RNA used as the template without the reverse transcription step (Fig. 6). These results support the existence of a long transcript starting from at least *xuaS* and covering all the genes up to *xuaJ.*

## Discussion

### A new AXOS uptake and degradation system in a primary degrader

**Fig. 7:**
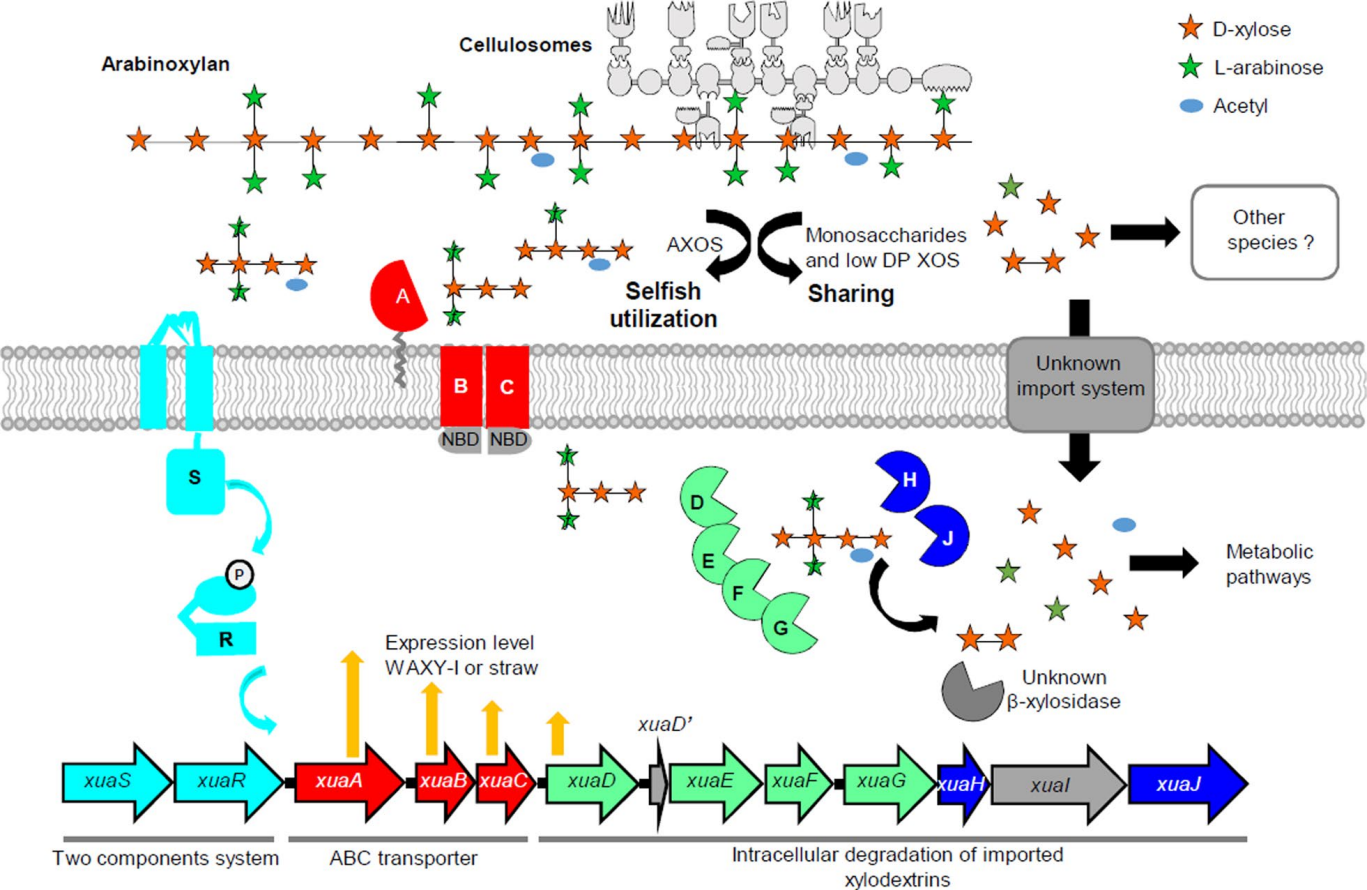
Model for arabinoxylan utilization by *R. cellulolyticum* in its natural biotope Cellulosomes depicted in light gray release large AXOS, short XOS and monosaccharides from arabinoxylan. A signal is transmitted from the sensor XuaS to the regulator XuaR (light blue) which subsequently induces the expression of the genes of the *xua* cluster (orange arrows). The ABC-transporter (red) imports a large range of AXOS from the environment in the cytoplasm, where GHs (light green) and esterases (dark blue) ultimately release arabinose, xylose and xylobiose. Xylobiose might be degraded by another unknown GH. Short XOS and monosaccharides might also be imported by *R. cellulolyticum* and other species found in the same biotope. Unknown function proteins are depicted in dark grey (XuaI, β-xylosidase and monosaccharide importer, NBD)

Our study shows that *R. cellulolyticum* can take up and intracellularly depolymerize arabinoxylan dextrins composed of at least 6 monosaccharides, as summarized in the model depicted in Fig. 7. During the growth on arabinoxylan, the secreted cellulosomes can release AXOS, XOS, arabinose, and xylose. AXOS and/or XOS might be the signal sensed by XuaS which, after phosphorylation of XuaR might induce the expression of the downstream genes encoding the ABC-transporter and the AXOS/XOS degrading cytosolic enzymes. Monosaccharides such as arabinose or xylose might not be involved in the signaling, since they do not induce the expression of the *xua* genes (Fig. 2). AXOS are imported by the bacterium through the Xua ABC-transporter energized with an unknown ATPase which is not encoded in the *xua* cluster as previously observed for other oligosaccharide ABC-importers in *R. cellulolyticum* [21, 29]. AXOS are further enzymatically degraded by the glycoside hydrolases XuaD, E, F, and G, with the help of the esterases XuaH and or J which ensure(s) the deacetylation of the imported oligosaccharides.

The SBP XuaA failed to bind to xylose, arabinose, and XOS but can interact with a large panel of mono and di-substituted AXOS of different sizes with similar *K*_D_ (12–63 nM). Similar uptake systems have been reported in a few bacteria with ABC-transporters varying in terms of specificity and affinity for the bound sugar. Some of the involved SBPs are specific to XOS [8, 17], and others can bind to both XOS and AXOS [9, 10]. Only the SBP BpAXBP2, from *Bifidobacterium pseudocatenulatum*, such as XuaA, is specific for AXOS, including double arabinosyl decorated ones, but with a preference for the shorter backbone (X3) and with a tenfold higher *K*_D_ (660 to 690 nM) [11]. Overall, XuaA binds with the highest affinity to the largest and most decorated AXOS. Acetylated AXOS which could not be tested, might also be imported when the bacterium grows on natural raw substrates. The presence of two acetyl esterases encoded in the *xua* cluster probably facilitates the action of GHs and permits the release of unacetylated monosaccharides from imported dextrins that can directly enter the metabolic pathways (Fig. 7). It is to be noted that most of the clusters described importing (arabino)xylo-oligosaccharides in other Gram-positive bacteria also contain genes predicted to encode esterases [7–10, 17], and two of these enzymes were biochemically characterized as acetyl esterases [10, 40]. Strikingly, the mix of Xua GHs depolymerizes AXOS into xylose, arabinose, and xylobiose as the final products. Xylobiose cannot be degraded by any of the enzymes encoded in the cluster and might be cleaved by a cytoplasmic enzyme encoded by a gene located elsewhere and possibly co-regulated. The enzymes encoded by the genes at loci Ccel_0203 (GH3), Ccel_1011 (GH43), Ccel_2614 (GH43) and Ccel_3438 (GH43) are potential candidates that will need to be examined.

*R. cellulolyticum* secretes cellulosomes and is a primary degrader able to grow on cellulose, arabinoxylan, and xyloglucan [20–22, 27, 32, 41]. When assayed on xyloglucan, the purified cellulosomes generate large xyloglucan dextrins containing up to 9 monosaccharides as the end product. The latter are further captured and imported by a specific ABC-transporter, which was shown to be essential for the growth of *R. cellulolyticum* on xyloglucan [21]. In contrast, when purified cellulosomes are incubated with arabinoxylan, they generate smaller end products, mainly arabinose, xylose, X2, and X3 [20, 32]. The genome of *R. cellulolyticum* encodes eight cellulosomal α-L-arabinofuranosidases/xylanases which contribute to the degradation of arabinoxylan by the secreted complexes. Moreover, other extracellular enzymes might participate in the degradation of (arabino)-xylan, since four genes are predicted to encode two enzymes in the free state (not assembled in the cellulosomes) and two cell-bound xylanases. These observations may explain why the growth of the *xuaA* mutant strain is slowed, but not abolished. As *R. cellulolyticum* was shown to grow on arabinose and xylose, it is highly likely that elemental monosaccharides released by cellulosomes (and other putative xylanases) during growth on arabinoxylan are imported by other transporters (Fig. 7), [41]. It is remarkable that none of the reported xylanolytic bacteria with a XOS/AXOS import system produce cellulosomes [8–12, 17]. The present study thus shows that such an import and intracellular degradation system is not limited to bacteria having a limited or non-existent xylanolytic system, and can be extended to plant cell wall degrading bacteria having an efficient extracellular degradation machinery like the cellulosomes.

**Fig. 8:**
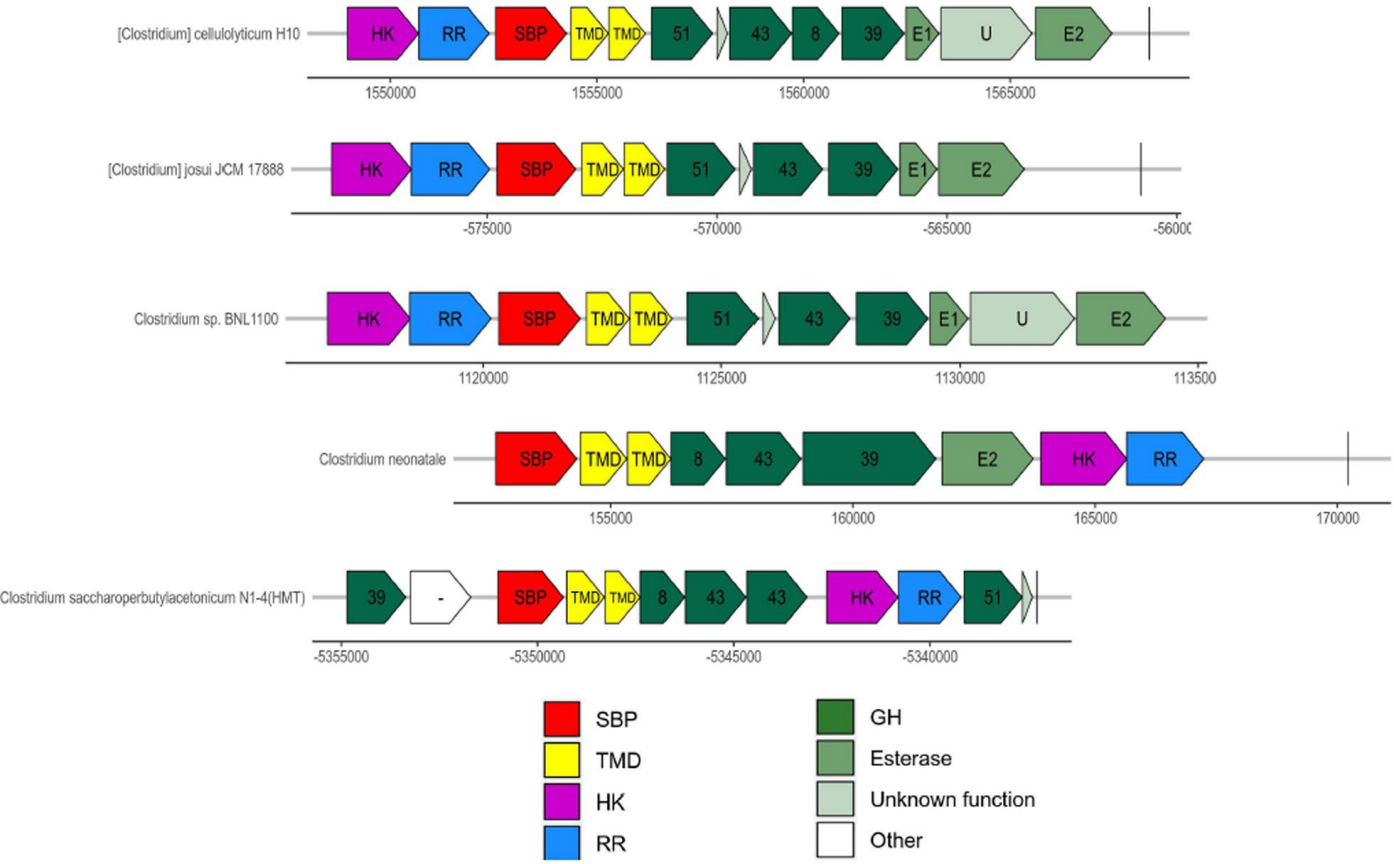
Synteny analysis of *xua* cluster The loci are aligned with respect to the AXOS specific solute binding protein XuaA (SBP) encoding genes. Other genes homologous to the *xua* gene cluster encode the transmembrane domain XuaB, C (TMD), the glycoside hydrolases XuaD, E, F, G (GH), the esterases XuaH and J (E1 and E2, respectively), proteins of unknown function (U) (XuaD’ and XuaI), and a two-component system including a sensor histidine kinase (HK) and a response regulator (RR). In white are found genes encoding for proteins different from the Xua proteins. Numbers in genes encoding GH refer to the GH family to which they belong

The *xua* cluster orthologs are found in a limited number of species, most of them being mesophilic firmicutes bacteria closely related to *R. cellulolyticum* (Fig. 8). Overall the predicted enzymatic equipment in these species is well-conserved but with variations in the synteny of the cluster of genes. The genes *xuaD’* and *xuaH and xuaI* for instance are not systematically present.

### Selfish versus extracellular hydrolysis of arabinoxylan

The new AXOS uptake and degradation system described in the present study allows the bacterium to import and secure oligosaccharides in the cytoplasm. It could be defined as a “selfish” system as proposed in the human gut Gram-negative *Bacteroides thetaiotaomicron* which can import mannodextrins and degrade them in the periplasm [42]. Similar systems were also described in Gram-positive bacteria for XOS and AXOS [7–13], and also for other types of polysaccharides, such as glucuronoxylan, arabinan or galactan, but in this case, the oligosaccharides are depolymerized directly in the cytosol [7, 14–17]. The “selfish” system of AXOS utilization in *R. cellulolyticum* is assumed to provide several advantages explaining the utility of this system in the metabolic strategy of the utilization of arabinoxylan. On one hand, importing large oligosaccharides composed of up to 6 monosaccharides is energy-saving, since it reduces the energy cost of the import per monosaccharide compared to the uptake of monosaccharides or shorter dextrins. This energy-saving system is especially important for obligate anaerobic cellulolytic bacteria growing with low yields of ATP supplied from catabolism. On the other hand, as importing monosaccharides is common to many bacteria, importing highly decorated oligosaccharides can confer a strong advantage that might be decisive for the survival of *R. cellulolyticum* in a competitive environment. The “selfish” uptake of *R. cellulolyticum* with its high-affinity SBP XuaA, might indeed rapidly hunt AXOS even at low concentrations, possibly as soon as they are released by either the cellulosomes found in the vicinity of the cells and/or the putative cell bound xylanases. The captured dextrins are then no longer available for other species, which are not able to capture them, nor for further extracellular break down in monosaccharides that could fuel other species.

In addition to this competitive advantage, it has to be noticed that the bacterium also might have a cooperative behavior, as suggested by the fact that the cellulosomes produced by the bacterium are not tethered to the cell envelope [22, 28]. They are hypothesized to diffuse in the nearby environment of the bacterium and to achieve complete depolymerization of AXOS and XOS, ultimately providing monosaccharides or low DP oligosaccharides to other surrounding species by cross-feeding (Fig. 7). Therefore, with high energy costs, the bacterium secretes cellulosomes whose depolymerizing activities might also benefit other non-hemicellulolytic microorganisms. In turn, *R. cellulolyticum* could benefit from the fact that the concentration of simple sugars is kept low thanks to their consumption by other bacteria, thus limiting the feedback inhibition of cellulosomal enzymes and increasing their hydrolytic efficiency. *R. cellulolyticum* could also utilize molecules of interest provided by other species, such as vitamins or fermentation products. Such cooperation between different species was reported in the gut, or in environmental biotopes [2, 19, 23, 43].

The primary degrader *R. cellulolyticum* thus seems to have developed both “selfish” and “sharing” strategies. The extracellular degradation of arabinoxylan by the bacterium leads to the release of fermentable carbohydrates of different sizes, such as arabinose, xylose, and low DP XOS/AXOS. Its “selfish” system bypasses direct competition for monosaccharides by securing large dextrins that only a few microorganisms might be able to import. The new “selfish” uptake system described in *R. cellulolyticum* thus appears to be the key to support its fitness and survival within anaerobic communities.

## Conclusion

This study describes a complete system for the import and intracellular degradation of the partial degradation product of arabinoxylan by a primary degrader. The AXOS imported via the Xua ABC transporter are depolymerized and deacetylated by the intracellular enzymes of the system. For the first time, direct genetic evidence reveals that part of the arabinoxylan depolymerization is achieved in the cell. This system is important for the fitness of the strain, even if it has an efficient extracellular enzymatic system that release monosaccharides in the extracellular space. These results provide new insight into the catabolic strategy encountered by some primary degraders and will help to design new synthetic microbial communities for the production of chemicals or biofuel from lignocellulosic biomass.

## Materials and methods

### Strains, media, and vectors

Strains and vectors used in this study are reported in Additional file 8. *Escherichia coli* strains were grown at 37 °C in Luria–Bertani medium supplemented with appropriate antibiotics (100 µg. mL^−1^ of ampicillin, 50 µg. mL^−1^ of kanamycin, or 35 µg. mL^−1^ chloramphenicol). *R. cellulolyticum* H10 ATCC 35319 and mutants were grown anaerobically at 32 °C on basal medium [30] supplemented with either 2 g. L^−1^ cellobiose, glucose, xylose, arabinose (Sigma-Aldrich, Darmstadt, Germany), wheat flour insoluble arabinoxylan, (WAXY-I), (Megazyme, Bray, Ireland); or 5 g.L^−1^ water washed hatched and grounded wheat straw prepared as previously described [44] (Valagro, Poitiers, France). Primers used in this study are reported in Additional file 9.

### Construction of *xuaA* mutant in *R. cellulolyticum*

The ClosTron method was used with electrotransformation to construct mutants as previously described using specific primers [28, 45]. The mutant strain interrupted in the gene located at the locus Ccel_1252 with group II intron was called MTL*xuaA*. Intron insertion was verified by PCR, and by Southern blot as previously described (Additional file 6) [46].

### Complementation of MTL *xuaA* mutant

The pSOSzeroTm was used as the control, and the vector pSOS956 was used to overexpress the genes *xuaABC* or *xuaABCD* (Additional file 8). PCR encompassing the genes *xuaABC* and *xuaABCD* from genomic DNA of *R. cellulolyticum* was carried out using specific primers (Additional file 9), amplicons and pSOS956 vector were digested with EheI and BamHI and ligated, resulting in pSOS*xuaABC*, pSOS*xuaABCD*.

### RNA preparation, reverse transcription

*R. cellulolyticum* was grown in minimal medium supplemented with arabinose, xylose, arabinoxylan (WAXY-I), or wheat straw until the mid-to-late-log exponential phase of growth, cells were harvested by centrifugation (7000 g, 5 min, 6 °C) then flash frozen in liquid N_2_ before storage at − 80 °C. Maxwell^®^ 16 miRNA Tissue Kit (Promega, Madison, USA) was used for total RNA isolation. Extra DNase treatment, RNA quality control and reverse transcription were performed as previously described [47].

### Transcriptional analyses

Quantitative real-time PCR was performed with specific primers on cDNA synthesized from mRNA as previously described (Additional file 9) [47]. For each point, technical duplicates and biological triplicates were performed. 16S RNA encoding gene was used as the reference for normalization, the amplification efficiencies for each primer pairs varied between 90% and 108%.

For Intergenic links, cDNA was synthesized from mRNA extracted from a culture of a WAXY-I grown *R. cellulolyticum*, using a specific primer to *xuaJ* (1262-S-RT-do) and the GoScript Reverse transcriptase (Promega) according to the manufacturer instructions. Amplifications with specific primers (Additional file 9) were performed with the PrimeSTAR GXL DNA polymerase (TaKaRa) on genomic DNA, cDNA, or mRNA, according to the manufacturer instructions.

### Cloning of the genes for recombinant protein production in *E. coli*

Recombinant mature proteins XuaA, XuaD’, XuaD, XuaE, XuaF, XuaG, XuaH, XuaI, and XuaJ were designed to contain 6 histidines residues at the N-terminus (XuaA) or at the C-terminus (others). The genes were amplified by PCR using gDNA from *R*. *cellulolyticum* as the matrix and the corresponding primers pair (Additional file 9). Amplicons were either digested with NdeI and XhoI or with NcoI and XhoI and ligated with NdeI-XhoI linearized pET22b (+) or with NcoI-XhoI linearized pET28b (+), respectively. After sequencing (Genewiz, Leipzig, Germany), the generated vectors were used to transform the BL21(DE3) strain (Invitrogen) to produce the recombinant proteins.

### Production and purification of the recombinant proteins

Purification was performed as previously described [30], except for the induction step performed at 20 °C for XuaA, and XuaF, and at 16 °C for the other proteins. The concentration of the purified proteins was determined from the absorbance at 280 nm and using the extinction coefficient calculated based on the basis of the primary sequence of each recombinant protein (https://web.expasy.org/protparam/). A small amount of proteolyzed protein was obtained from BL21(DE3) strains producing XuaI, and no protein could be purified for XuaD’, thus preventing their enzymatic characterization.

### SDS–PAGE and Western blot analysis

SDS–PAGE and western blot were performed as previously described [28]. Cells were grown to the mid-exponential stage of growth in minimum medium containing glucose. An equivalent of 2 ml of culture at OD 0.5 (450 nm) was centrifuged (7000 g, 15 min, 4 °C) and suspended in 40 µl of SDS loading buffer. 10 µl of the sample was loaded in SDS–PAGE for Western blot analysis. Primary antibody was obtained from an immunized rabbit using purified XuaA (ProteoGenix, Schiltigheim, France).

### Thermal shift assay (TSA) and isothermal titration calorimetry (ITC)

XOS and AXOS were purchased from Megazyme: A^3^X (ref O-A^3^X), A^2^XX (ref O-A^2^XX), A^2,3^XX (ref O-A^2,3^XX), XA^3^XX (ref O-XA^3^XX), XA^2,3^XX (ref O-XA^2,3^XX), XAXXMIX (ref O-XAXXMIX), X2 to X6 (ref O-XBI, O-XTR, O-XTE, O-XPE, O-XHE, respectively) (Fig. 3) (Megazyme). For TSA experiments, monosaccharides, XOS and AXOS were tested at a final concentration of 1 mM or 0.1 mM (for X4, X5, and X6). Experiments were performed with XuaA (8 µM) in 25 mM phosphate buffer pH 7 and SYPRO orange (1/500) (Sigma-Aldrich). Melting curves were monitored from 10 to 90 °C with 0.5 °C increment every 30 s, on CFX96 touch real-time PCR detection system (Bio-Rad, France) and analyzed using the Bio-Rad CFX Maestro 1.1 software.

A MicroCal PEAQ-ITC (Malvern, Malvern, UK) was used to determine thermodynamic parameters of the binding at 25 °C in 25 mM phosphate buffer, pH 7.0. The ligands (50–2000 µM) were injected (one injection with 0.4 µL followed by 18 injections of 2 µL) in the cell containing XuaA (5–50 µM) in the same buffer. At least one duplicate was performed.

### Enzyme activity measurement

XuaD, E, F, G (0.01 to 1 µM) were tested with different AXOS and/or XOS at 1 mM with incubation times varying from 5 min to 24 h. Enzymes (10 nM) were incubated at 37 °C for 5 min, kinetic parameters of XuaD were determined with both A^2,3^XX and A^2^XX, with sugar concentration varying from 0.05 to 8 mM, and XuaE with A^2,3^XX with sugar concentration varying from 0.05 to 1 mM (at least 9 concentration point). Substrates and released products were quantified by high performance anion exchange chromatography coupled with pulsed amperometric detection on an ICS-3000 using a PA1 column (Thermo Scientific). Sugars were eluted with the buffers 0.1 M NaOH and 0.5 M sodium acetate + 0.1 M NaOH as the eluants A and B, respectively, using the multistep procedure as follows: isochratic separation (5 min, 95% A + 5% B), separation gradient (8 min, 10–37% B), column wash (2 min, 99% B), and subsequent column equilibration (2.5 min, 95% A + 5% B).

Esterase activity was tested using 1-Naphthyl acetate. Release of 1-Naphthol was monitored at 320 nm during 5 min at 37 °C in 25 mM phosphate buffer pH 7, with substrate final concentration ranging from 110 µM and 5.36 mM, and with XuaH (0.1 µM) or XuaJ (0.01 µM). Concentration of released 1-Naphthol during the first minute of the assay was determined based on 1-Naphthol standard curve.

### Bioinformatic analysis

We looked for species that present a syntenic region to the *R. cellulolyticum xua* cluster. From the 5609 bacterial genomes of OrthoDB v10 we extracted the homologous genes of each of the 13 *xua* genes of the bacterial orthologous groups defined by OrthoDB. The corresponding genomic annotation files downloaded from the NCBI server were scanned sequentially by an internal python script to identify regions containing at least 4 genes belonging to the same orthologous groups as the *xua* genes coding enzymes (XuaD, E, F, G, H, J) or hypothetical protein (XuaI). The genes must have a distance of less than 10 000 bp from each other. Each region is extended to include homologous genes to the other *xua* genes (XuaS, R, A, B, C) with the same distance criterium. The conservation of the gene order is observed afterward.

Protein sequences were analyzed using online program, PFAM (http://pfam.xfam.org/) [48] and ESTHER database (http://bioweb.ensam.inra.fr/esther) [49], Carbohydrate Active Enzymes database (http://www.cazy.org/) [6].

## Supplementary Information

**Additional file 1.** Interaction of XuaA with various carbohydrate ligands using thermal shift assay is presented.

**Additional file 2.** Interaction of XuaA with various carbohydrates using isothermal titration calorimetry is presented.

**Additional file 3.** Analysis of the digestion products released by XuaD and XuaE from AXOS. Chromatograms obtained after High Pressure Anion Exchange Chromatography coupled with Pulsed Amperometric Detection (HPAEC–PAD) are presented.

**Additional file 4.** Analysis of the digestion products released by XuaF and XuaG from AXOS and XOS. Chromatograms obtained after High Pressure Anion Exchange Chromatography coupled with Pulsed Amperometric Detection (HPAEC–PAD) are presented.

**Additional file 5.** Kinetic analysis of XuaD, XuaE, XuaH and XuaJ with various ligands are presented.

**Additional file 6.** Molecular analysis of the *Ruminiclostridium* *cellulolyticum *mutant strain. PCR and southern blot analysis on the wild-type and the mutant strains are presented.

**Additional file 7.** Growth of *R*. *cellulolyticum* wild-type, mutant and derivatives strains on arabinose. Growth curves are presented.

**Additional file 8.** Strains and vectors used in this study. Table with strains and vectors used in this study and their relevant characteristics and sources and references are presented.

**Additional file 9.** Primers used in this study. Table with the primers used in the study and their sequence are presented.

## Data Availability

All data generated or analyzed during this study are included in this published article and its additional files.

## Abbreviations

SBP: Solute binding protein
TMD: Transmembrane domain
NBD: Nucleotide binding domain
GH: Glycoside hydrolase
CE: Carbohydrate esterase
AXOS: Arabinoxylo-oligosaccharide
XOS: Xylo-oligosaccharide
