# Supplementary material for: Selfish uptake versus extracellular arabinoxylan degradation in the primary degrader Ruminiclostridium cellulolyticum, a new string to its bow
Source: Biotechnol Biofuels Bioprod. 2022 Nov 19;15:127. doi: 10.1186/s13068-022-02225-8 (PMC9675976; doi:10.1186/s13068-022-02225-8)
Supplement: Supplementary file 1 — Additional file 1. Interaction of XuaA with various carbohydrate ligands using thermal shift assay is presented. [file 13068_2022_2225_MOESM1_ESM.pdf]

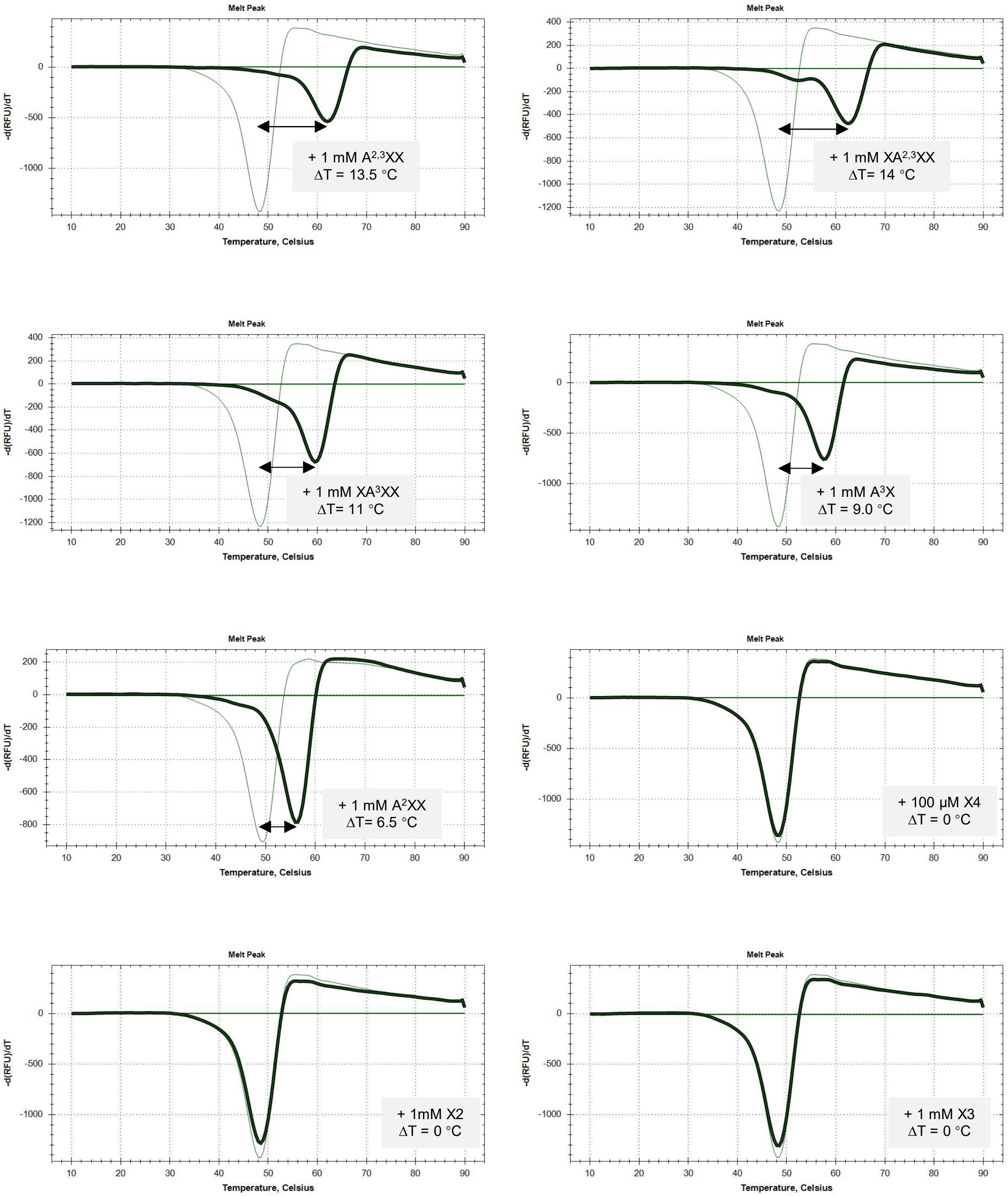
Melt Peak
-d(RFU)/dT
Temperature, Celsius
+ 1 mM A2,3XX
ΔT = 13.5 °C
Melt Peak
-d(RFU)/dT
Temperature, Celsius
+ 1 mM XA2,3XX
ΔT= 14 °C
Melt Peak
-d(RFU)/dT
Temperature, Celsius
+ 1 mM XA3XX
ΔT= 11 °C
Melt Peak
-d(RFU)/dT
Temperature, Celsius
+ 1 mM A3X
ΔT = 9.0 °C
Melt Peak
-d(RFU)/dT
Temperature, Celsius
+ 1 mM A2XX
ΔT= 6.5 °C
Melt Peak
-d(RFU)/dT
Temperature, Celsius
+ 100 μM X4
ΔT = 0 °C
Melt Peak
-d(RFU)/dT
Temperature, Celsius
+ 1mM X2
ΔT = 0 °C
Melt Peak
-d(RFU)/dT
Temperature, Celsius
+ 1 mM X3
ΔT = 0 °C

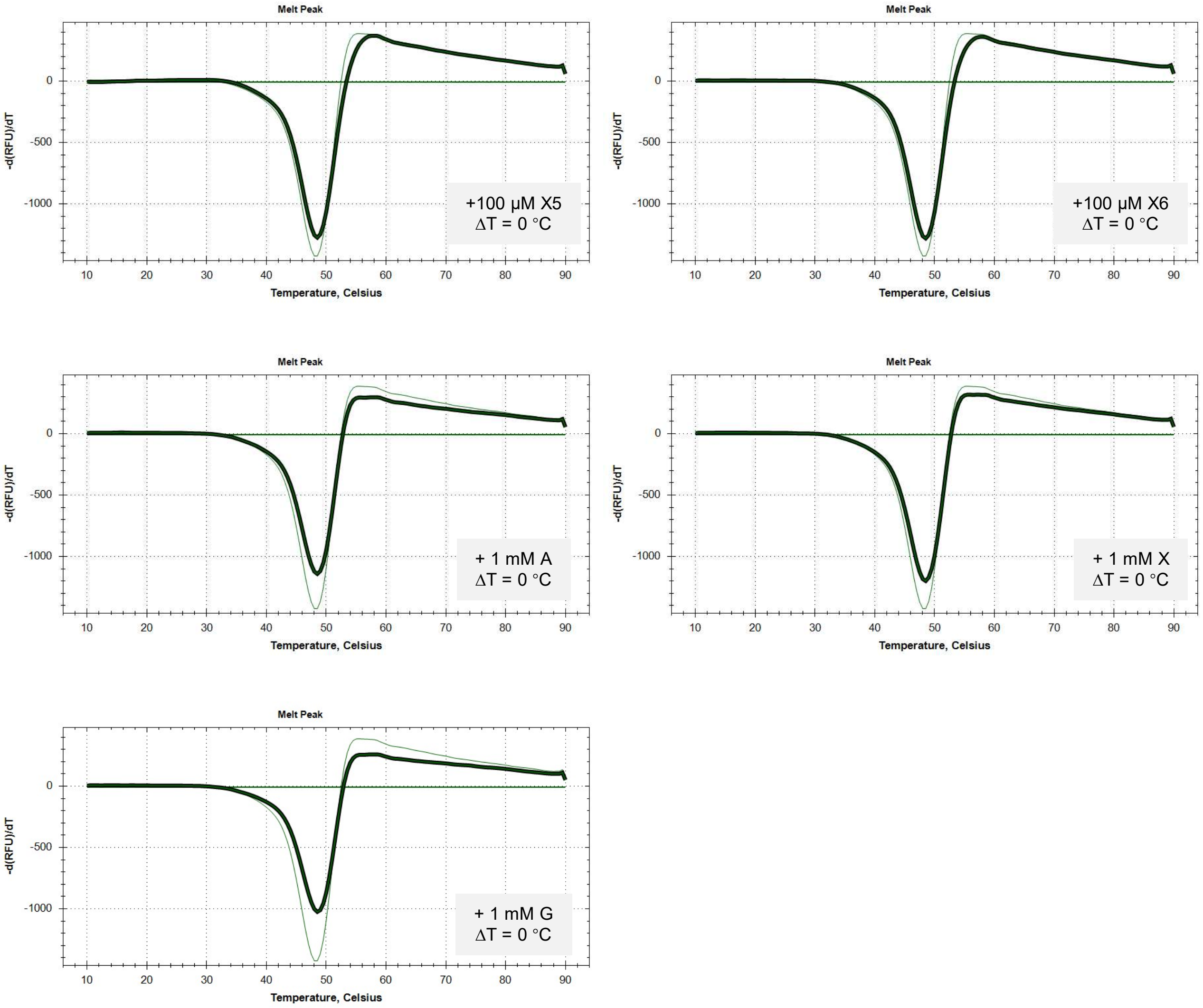

Additional file 1. Interaction test between XuaA and various carbohydrate by thermal shift assay. XuaA was incubated with the sugars in presence of SYPRO orange and the temperature was increased from 10 to 90°C with 0,5°C increments every 30 s. The melting curve obtained corresponds to the first derivative of the fluorescence (Raw Fluorescence Unit) plotted as a function of the temperature. Green, the melting curve of XuaA without ligand, black bold, the melting curve of XuaA with the tested sugars as indicated. All graphs are representative of three experiments. The shift is visualized with the two-heads arrows. X, A and G are xylose, arabinose and glucose, respectively.
