# Supplementary material for: Selfish uptake versus extracellular arabinoxylan degradation in the primary degrader Ruminiclostridium cellulolyticum, a new string to its bow
Source: Biotechnol Biofuels Bioprod. 2022 Nov 19;15:127. doi: 10.1186/s13068-022-02225-8 (PMC9675976; doi:10.1186/s13068-022-02225-8)
Supplement: Supplementary file 2 — Additional file 2. Interaction of XuaA with various carbohydrates using isothermal titration calorimetry is presented. [file 13068_2022_2225_MOESM2_ESM.pdf]

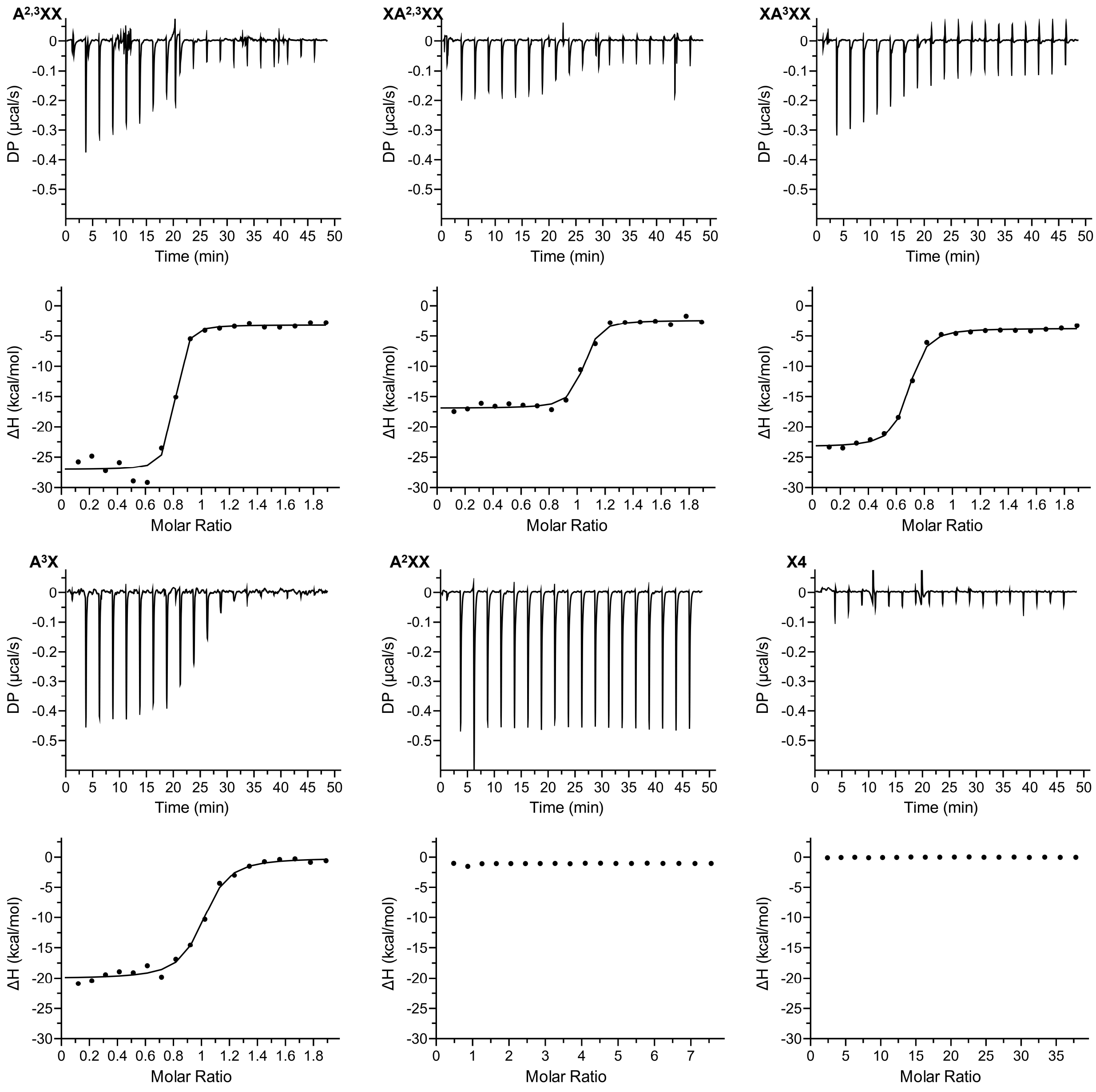

Additional file 2. Isothermal titration calorimetry of XuaA and various carbohydrates

Integrated binding heats of interactions of XuaA with various carbohydrates. XuaA is in the cell and ligand in the syringe.
