# Supplementary material for: Selfish uptake versus extracellular arabinoxylan degradation in the primary degrader Ruminiclostridium cellulolyticum, a new string to its bow
Source: Biotechnol Biofuels Bioprod. 2022 Nov 19;15:127. doi: 10.1186/s13068-022-02225-8 (PMC9675976; doi:10.1186/s13068-022-02225-8)
Supplement: Supplementary file 3 — Additional file 3. Analysis of the digestion products released by XuaD and XuaE from AXOS. Chromatograms obtained after High Pressure Anion Exchange Chromatography coupled with Pulsed Amperometric Detection (HPAEC–PAD) are presented. [file 13068_2022_2225_MOESM3_ESM.pdf]

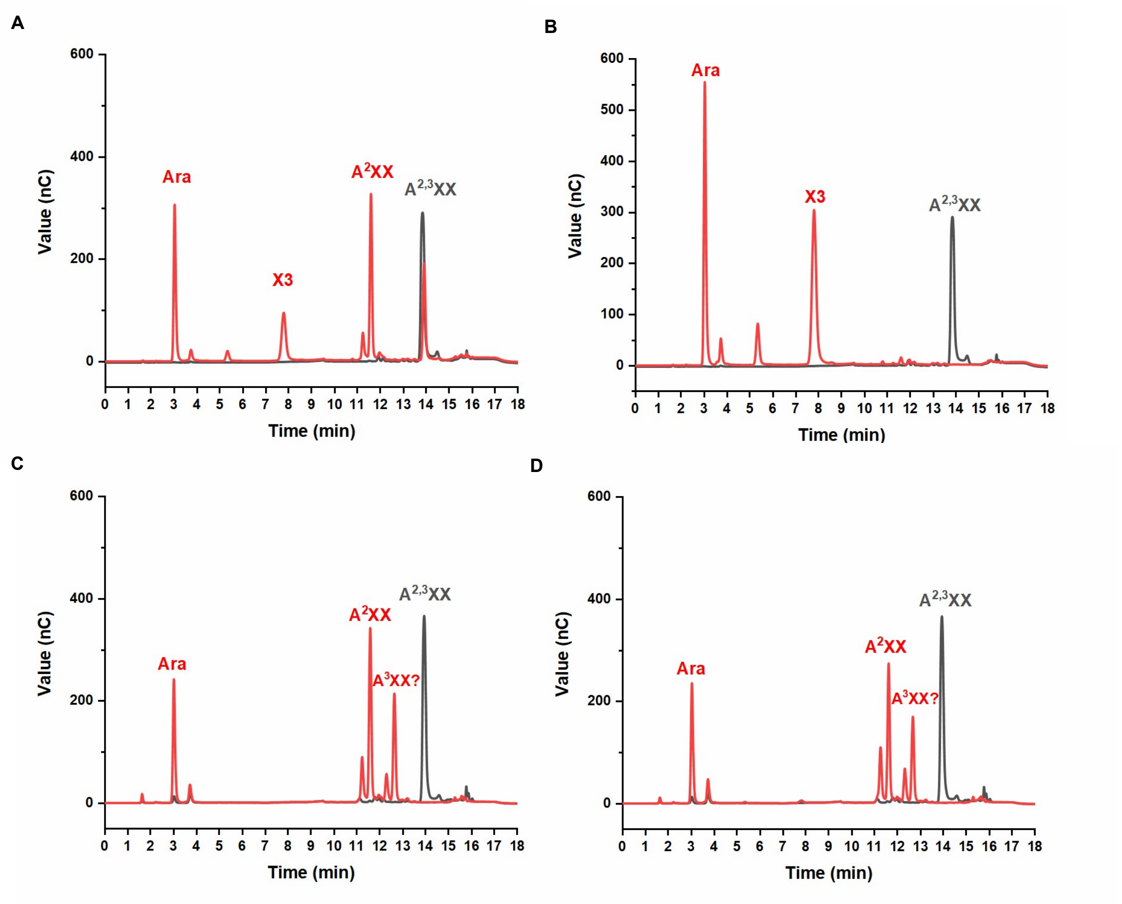

Additional file 3. Analysis of the digestion products released by XuaD and XuaE from AXOS.
Chromatograms obtained after High Pressure Anion Exchange Chromatography coupled with Pulsed Amperometric Detection (HPAEC-PAD) are presented. Analysis of the substrate before and after enzyme treatment are shown in dark gray and red respectively. XuaD (100 nM) was incubated with $A^{2,3}XX$ 1 mM at 37 °C for 5 min (A) and 30 min (B); XuaE (1 µM) was incubated with $A^{2,3}XX$ 1 mM at 37 °C for 10 min (C) and 24 h(D).
