# Supplementary material for: Selfish uptake versus extracellular arabinoxylan degradation in the primary degrader Ruminiclostridium cellulolyticum, a new string to its bow
Source: Biotechnol Biofuels Bioprod. 2022 Nov 19;15:127. doi: 10.1186/s13068-022-02225-8 (PMC9675976; doi:10.1186/s13068-022-02225-8)
Supplement: Supplementary file 4 — Additional file 4. Analysis of the digestion products released by XuaF and XuaG from AXOS and XOS. Chromatograms obtained after High Pressure Anion Exchange Chromatography coupled with Pulsed Amperometric Detection (HPAEC–PAD) are presented. [file 13068_2022_2225_MOESM4_ESM.pdf]

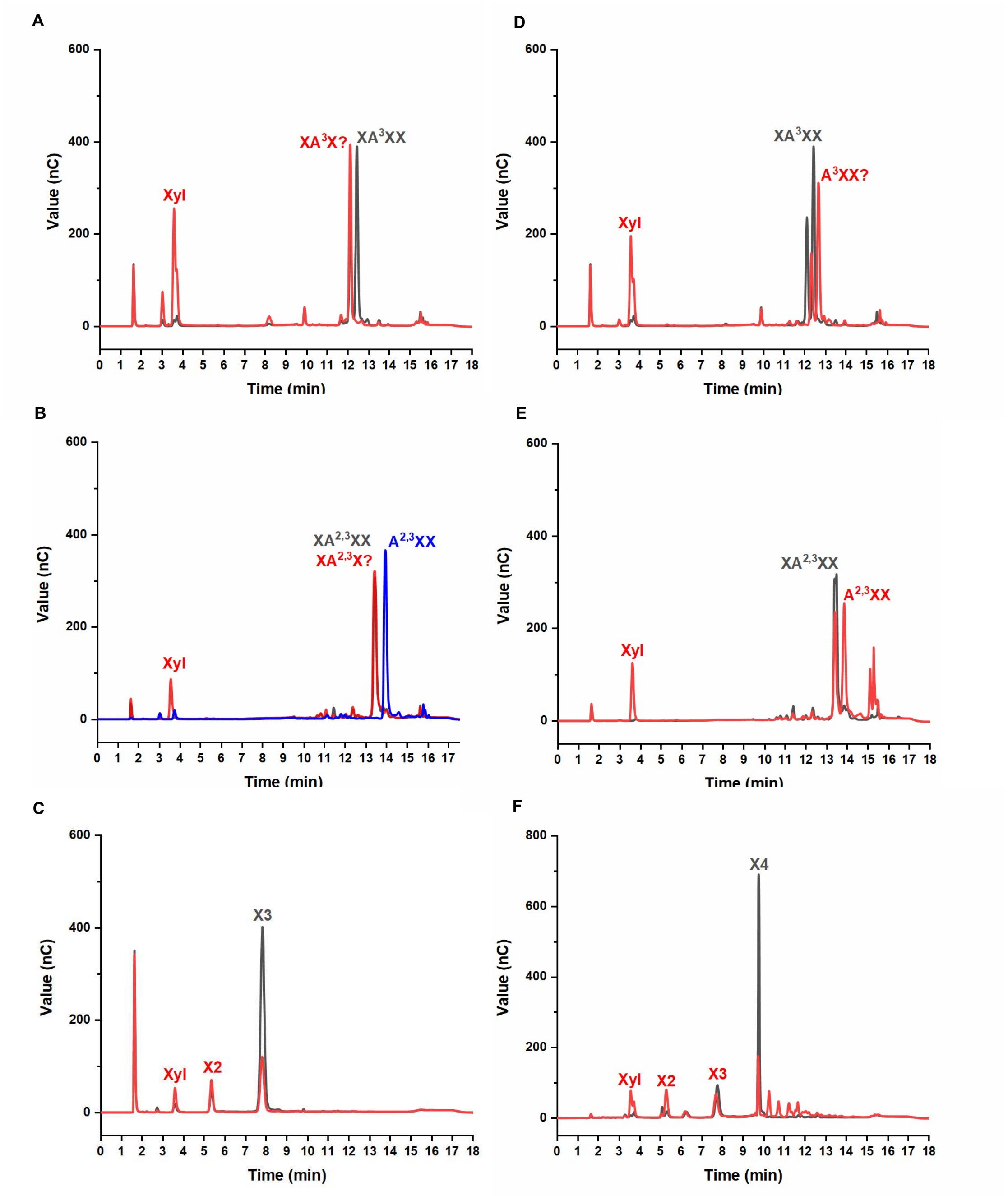

Additional file 4. Analysis of the digestion products released by XuaF and XuaG from AXOS and XOS. Chromatograms were obtained after High Pressure Anion Exchange Chromatography coupled with Pulsed Amperometric Detection (HPAEC-PAD). Analysis of the substrate before and after enzyme treatment are shown in dark gray and red respectively. XuaF (1 µM) was incubated with XA^3XX 1 mM at 37 °C for 24 h (A) or $XA^{2,3}XX$ (B) 1 mM at 37 °C for 10 min respectively; For the panel B the chromatogram of the reference $A^{2,3}XX$ (blue) has been overlaid with the substrate and the product; XuaF (0.1 µM) was incubated with X3 1 mM at 37 °C for 24 h (C); XuaG (1 µM) was incubated with XA^3XX 1mM at 37 °C for 24 h (D) , $XA^{2,3}XX$ 1 mM at 37 °C for 10 min (E) or X4 at 37 °C for 10 min (F).
