# Supplementary material for: Selfish uptake versus extracellular arabinoxylan degradation in the primary degrader Ruminiclostridium cellulolyticum, a new string to its bow
Source: Biotechnol Biofuels Bioprod. 2022 Nov 19;15:127. doi: 10.1186/s13068-022-02225-8 (PMC9675976; doi:10.1186/s13068-022-02225-8)
Supplement: Supplementary file 5 — Additional file 5. Kinetic analysis of XuaD, XuaE, XuaH and XuaJ with various ligands are presented. [file 13068_2022_2225_MOESM5_ESM.pdf]

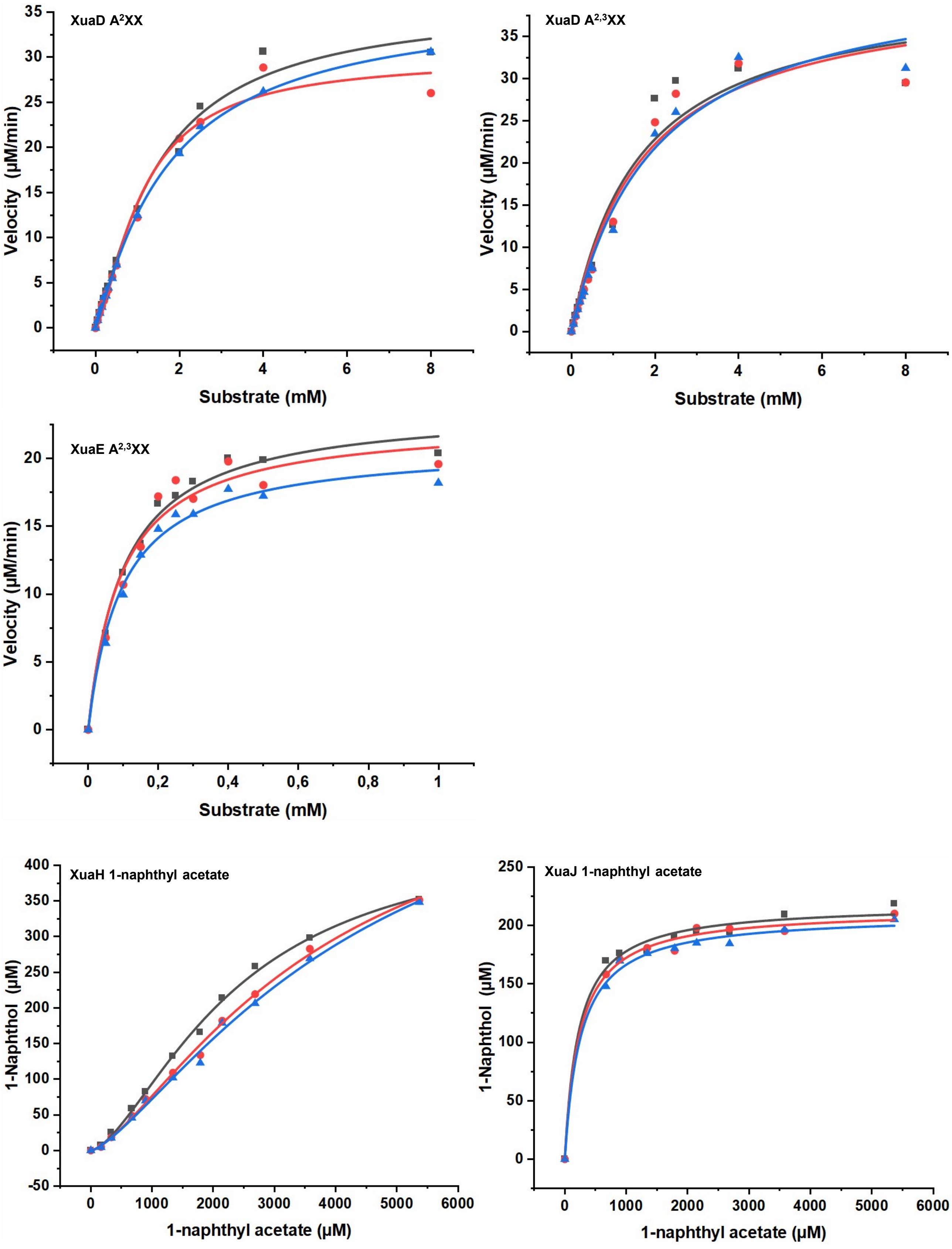

Additional file 5. Enzymatic kinetics for XuaD, E, H and J.

For XuaD and E, the velocity of arabinose release is plotted as a function of the concentration of the substrate as indicated. For XuaH and J the 1-Naphthol released is plotted as a function of the concentration of the substrate (1-Naphthyl acetate).
