# Supplementary material for: Selfish uptake versus extracellular arabinoxylan degradation in the primary degrader Ruminiclostridium cellulolyticum, a new string to its bow
Source: Biotechnol Biofuels Bioprod. 2022 Nov 19;15:127. doi: 10.1186/s13068-022-02225-8 (PMC9675976; doi:10.1186/s13068-022-02225-8)
Supplement: Supplementary file 6 — Additional file 6. Molecular analysis of the Ruminiclostridium cellulolyticum mutant strain. PCR and southern blot analysis on the wild-type and the mutant strains are presented. [file 13068_2022_2225_MOESM6_ESM.pdf]

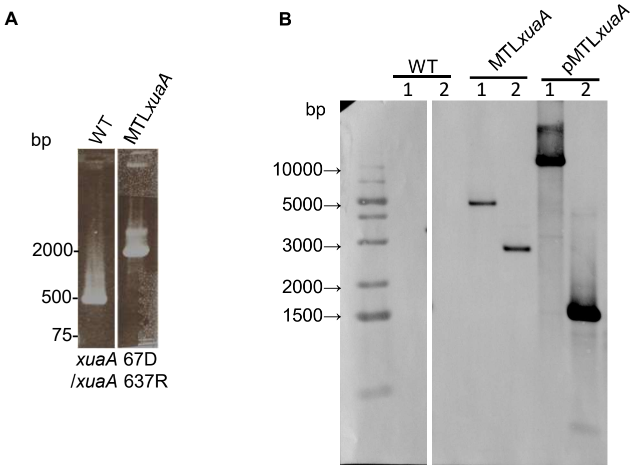

Additional file 6. Molecular analysis of the *Ruminiclostridium cellulolyticum* mutant strain

A. PCR analysis of WT gDNA and MTL*xuaA* strain cells using the primer pair *xuaA* 67D / *xuaA* 637R hybridizing upstream and downstream of the targeted type II intron insertion site in *xuaA*. Expected size of the amplicons are 590 bp in WT and 2371 bp when type II intron is inserted in *xuaA* in the *MTLxuaA* mutant strain.

B. Southern blot. WT and MTL*xuaA* mutant gDNA or pMTL*xuaA* were cut with HindIII (1) or HaeIII (2). After migration and transfert, the membrane was incubated with a labeled probe targeting erythromycin resistance cassette. The size of the detected fragments in MTL*xuaA* is consistent with theoretical one (4616 bp with HindIII and 2724 bp with HaeIII). Only one insertion is detected.
