# Supplementary material for: Selfish uptake versus extracellular arabinoxylan degradation in the primary degrader Ruminiclostridium cellulolyticum, a new string to its bow
Source: Biotechnol Biofuels Bioprod. 2022 Nov 19;15:127. doi: 10.1186/s13068-022-02225-8 (PMC9675976; doi:10.1186/s13068-022-02225-8)
Supplement: Supplementary file 7 — Additional file 7. Growth of R. cellulolyticum wild-type, mutant and derivatives strains on arabinose. Growth curves are presented. [file 13068_2022_2225_MOESM7_ESM.pdf]

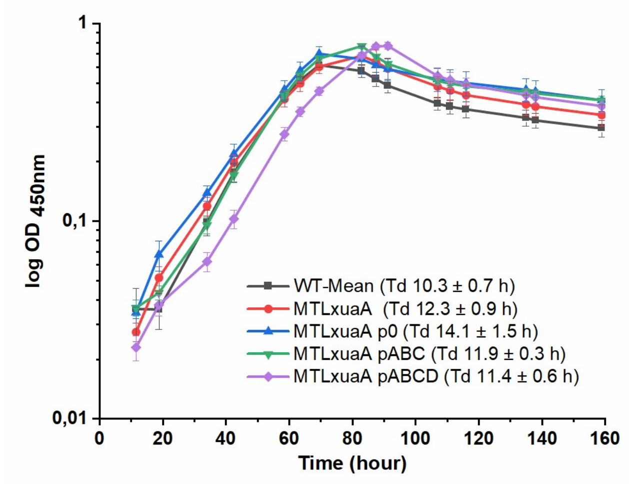

Additional file 7. Growth of *R. cellulolyticum* wild-type, mutant and derivatives strains on arabinose

The strains were grown on minimal medium containing 2 g L^{-1} arabinose, WT (gray), mutant strains MTL*xuaA* (red), MTL*xuaA* strain carrying an empty vector pSOSzeroTm (blue), pSOS*xua*ABC (green) or pSOS*xua*ABCD (purple). Experiments were performed in triplicates and bars indicate the standard deviation.
