# Supplementary material for: Selfish uptake versus extracellular arabinoxylan degradation in the primary degrader Ruminiclostridium cellulolyticum, a new string to its bow
Source: Biotechnol Biofuels Bioprod. 2022 Nov 19;15:127. doi: 10.1186/s13068-022-02225-8 (PMC9675976; doi:10.1186/s13068-022-02225-8)
Supplement: Supplementary file 8 — Additional file 8. Strains and vectors used in this study. Table with strains and vectors used in this study and their relevant characteristics and sources and references are presented. [file 13068_2022_2225_MOESM8_ESM.pdf]

Additional file 8. Strains and vectors used in this study

Strain	Relevant characteristics	Source or reference
E.coli NEB5α	*fhuA2* Δ(*argF*-*lacZ*)*U*169 *phoA glnV*44 *Φ*80 Δ(*lacZ*)M15 *gyrA96* recA1 *relA1 endA1 thi-1 hsdR17*	New England Biolabs
E.coli BL21(DE3)	F^- *ompT hsdSB* (rB^-mB^-) *gal dcm* (DE3)	Invitrogen
E. coli SG13009 (pREP4)	F- *his pyrD* D*lon-100 rpsL* (pREP4)	Qiagen
R. cellulolyticum	Wild-type, H10, ATCC35319, DSM 5812	DSMZ
R. cellulolyticum MTL*xuaA*	*xuaA* ::intron, Erm^r	This study
**Vector**	**Relevant characteristics**	**Source or reference**
pET22b(+)	*E. coli* expression vector, Amp^r	Novagen
pET28b(+)	*E. coli* expression vector, Km^r	Novagen
pET*xuaA*	pET22b(+) derivative carrying the NdeI-XhoI fragment encoding mature XuaA	This study
pET*xuaD*	pET22b(+) derivative carrying the NdeI-XhoI fragment encoding XuaD	This study
pET*xuaD'*	pET28b(+) derivative carrying the NcoI-XhoI fragment encoding XuaD'	This study
pET*xuaE*	pET28b(+) derivative carrying the NcoI-XhoI fragment encoding XuaE	This study
pET*xuaF*	pET28b(+) derivative carrying the NcoI-XhoI fragment encoding XuaF	This study
pET*xuaG*	pET28b(+) derivative carrying the NcoI-XhoI fragment encoding XuaG	This study
pET*xuaH*	pET22b(+) derivative carrying the NdeI-XhoI fragment encoding XuaH	This study
pET*xuaI*	pET28b(+) derivative carrying the NcoI-XhoI fragment encoding XuaI	This study
pET*xuaJ*	pET28b(+) derivative carrying the NcoI-XhoI fragment encoding XuaJ	This study
pMTL007	*E. coli* /*Clostridium* shuttle vector (ColE1, pCB102)Ll.*ltrB* intron (*erm* BtdRAM2) under the control of P*fac* , *ltr* A; Cm^r/Tm^r	Heap *et al* . 2007
pMTL*xuaA*	pMTL007 derivative targeting *xuaA* (locus Ccel_1252)	This study
pSOSzeroTm	*E. coli* /*Clostridium* shuttle vector (ColE1, pIM13); Ap^r, Cm^r/Tm^r	Celik *et al,* 2013
pSOS*xuaABC*	pSOS956 *E. coli* /*Clostridium* shuttle vector (ColE1, pIM13); Ap^r, Cm^r/Tm^r, derivative, carrying the BamHI-EheI fragment encoding full length *xuaA* to *xuaC* , under the control of a weakened *Clostridium acetobutylicum* thiolase gene promotor	Liu *et al* , 2019, this study
pSOS*xuaABCD*	pSOS956 *E. coli* /*Clostridium* shuttle vector (ColE1, pIM13); Ap^r, Cm^r/Tm^r, derivative, carrying the BamHI-EheI fragment encoding full length *xuaA* to *xuaD* , under the control of a weakened *Clostridium acetobutylicum* thiolase gene promotor	Liu *et al* , 2019, this study

Ap^r, ampicilline resistance; Km^r, kanamycin resistance, Erm^r, erythromycin resistance; Cm^r/Tm^r, chloramphenicol/thiamphenicol resistance
