# Supplementary material for: Selfish uptake versus extracellular arabinoxylan degradation in the primary degrader Ruminiclostridium cellulolyticum, a new string to its bow
Source: Biotechnol Biofuels Bioprod. 2022 Nov 19;15:127. doi: 10.1186/s13068-022-02225-8 (PMC9675976; doi:10.1186/s13068-022-02225-8)
Supplement: Supplementary file 9 — Additional file 9. Primers used in this study. Table with the primers used in the study and their sequence are presented. [file 13068_2022_2225_MOESM9_ESM.pdf]

Additional file 9. Primers used in this study

Name	Sequence	Note
xuaA _NdeI_Dir	AAAAAACAT**ATG**CACCACCACCACCACCACTGCGGTAGTCAAAACAATACATC	Production in *E. coli*
xuaA _XhoI_Rev	AAAATTCTCGAGTTACTTGGATTTAACTGAGTCATACC	
MG*xuaD* _NdeI_Dir	GTTTAACTTTAAGAAGGAGATATACAT**ATG**GTAAAAATGATTTTAAATGCAGATA	
MG*xuaD* _XhoI_Rev	ATCTCAGTGGTGGTGGTGGTGGTGCTCGAGTACAATTTCAAGCAATACAACTGATTTAG	
xuaDbis _NcoI_Dir	AAAAAACC**ATG**GGCAAGGATAATTGCTTAAAATGTCATAGA	
xuaDbis _XhoI_Rev	AAAAAACTCGAGCCACTTGTTGCCAGACG	
xuaE _NcoI_Dir	AAAAAACC**ATG**GGCAAAATTCAGAACCCCATTATATGG	
xuaE _XhoI_Rev	AAAATTCTCGAGTTGTCGTGATTCATAAGAAAAATTTCTG	
xuaF _NcoI_Dir	TTTAAACC**ATG**GGCTCAAAAGGAGCTTATTTTACAAAACAG	
xuaF _XhoI_Rev	TTTTTTCTCGAGGATGTAAATTTTATATTTTCCTGCCAGC	
MG*xuaG* _NcoI_Dir	TTAACTTTAAGAAGGAGATATACC**ATG**GGCAACAAAATAAAGATTTCAGATAAGGTTAC	
MG*xuaG* _XhoI_Rev	ATCTCAGTGGTGGTGGTGGTGGTGCTCGAGATACCCGGGAATCAAACTGTC	
xuaH _NdeI_Dir	TTTTTTCAT**ATG**GCAATTTTTCAAGCGAATTTT	
xuaH _XhoI_Rev	TTTTTTCTCGAGTTCAGTAGCCCAAGCAAGTC	
MG*xuaI* _NcoI_Dir	TTAACTTTAAGAAGGAGATATACC**ATG**GGCAAGCTGATATGCGGCGAC	
MG*xuaI* _XhoI_Rev	ATCTCAGTGGTGGTGGTGGTGGTGCTCGAGTAAACTATCAACATAATTAGCGTATATTC	
xuaJ _NcoI_Dir	AAAAAACC**ATG**GGCAATTCTAATCGCCCTAAAATAAGATTAC	
xuaJ _XhoI_Rev	AAAAAACTCGAGCTCCGTTGTAAAAGGTGAAGC	
qPCR_16s_dir	CTATGTTTCTTGAGTGCCGG	qRT-PCR
qPCR_16s_rev	ATACTTATTGTGTTAACTCCGG	
qPCR_*xuaS* _431_dir	CGGCAGGATTAAAGGGAGCA	
qRT_*xuaS* _708_rev	CCCATCACGATCCCGTTCTT	
qPCR_*xuaR* _277_dir	AAAGCAATTCAGCACCAGGG	
qPCR_*xuaR* _560_rev	CCAAGCAGCAGAATTACAGGA	
qPCR_*xuaA* _2nd_378_dir	ATCTGGAGAACTCCCTGATGT	
qPCR_*xuaA* _2nd_657_rev	AGTCTTAGGTGCCTGTAGGT	
qPCR_*xuaB* _346_dir	CCGCACTTTTTATCATGGGTTG	
qPCR_*xuaB* _584_rev	GCTGCTTCGTATAAACTGGGG	
qPCR_*xuaC* _429_dir	ATGGGCTTTGGTTCTTCCAG	
qPCR_*xuaC* _638_rev	CAGCTGTTCCAGTGACCTAC	
qPCR_*xuaD* _397_dir	CATGAAATGCAGCAGTGGGT	
qPCR_*xuaD* _647_rev	TCATCTACCGAAGCACCTCC	
qPCR_*xuaD'* _72_dir	AAGGGTAGTTAAATCGGGCG	
qPCR_*xuaD'* _254_rev	TACCACTTGTTGCCAGACG	
qPCR_*xuaE* _335_dir	GCGGTTGGGAACGTTATGTG	
qPCR_*xuaE* _619_rev	CAACACGCCTTCTGCTGTT	
qPCR_*xuaF* _293_dir	ACGAGGGAAAGTATCAGGGC	
qPCR_*xuaF* _524_rev	TCCCACATAGGTTCTCCACC	
qPCR_*xuaG* _297_dir	GCTGGGATTTATGCCGTCAC	
qPCR_*xuaG* _523_rev	ATTCGGACTCATCGGCATCC	
qPCR_*xuaH* _169_dir_2nd_	ACGGATTGGTTGACATGGAC	
qPCR_*xuaH* _403_rev_2nd	TTATTGCACCGTAACCACCC	
qPCR_*xuaI* _280_dir	GTAAAGGTGCTGAAGGCTGG	
qPCR_*xuaI* _560_rev	GACCGCATTGTTACATCCGA	
qPCR_*xuaJ* _222_dir_2nd	TGAAACGGAAGAGTCCCTTGA	
qPCR_*xuaJ* _447_rev_2nd	CTCCCAGATTCCTGCTTCCA	
xuaA -98a-EBS1d	CAGATTGTACAAATGTGGTGATAACAGATAAGTCCTGTCGGATAACTTACCTTTCTTTGT	Targeted mutagenesis
xuaA -98a-IBS	AAAAAAGCTTATAATTATCCTTAGCTGTCCTGTCGGTGCGCCCAGATAGGGTG	
xuaA -98a-EBS2	TGAACGCAAGTTTCTAATTTCGATTACAGCTCGATAGAGGAAAGTGTCT	
EBS universal primer	CGAAATTAGAAACTTGCGTTCAGTAAAC	
1252_BamHI_dir	GGGCGCGGATCCATTAGGAGGAAACAAATGAGATTCAATTAC	Complementation studies
1252_t153c_rev	TTTCCGAAAGGGTCCTTATTTGCTACTG	
1252_t153c_dir	CTACTTCTTCAGTAGCAAATAAGGACCCTTTC	
1254_NarI_rev	TTATGGCGCCTTATCCTTTTACACCACCGAGAAC	
1255_NarI_rev	CGGCGCGGCGCCTTATACAATTTCAAGCAATACAACTGATTTAG	
ORF-up	GGGGCTCGAGGCTCCTTGGAATACATAGAA	Check pMTL vector
ORF-do	GGGGCGATCGTACTGTGTCGGCACTCTCAA	
xuaA 67D	GTTGTAGCTTGCGGTAGTC	Check intron insertion in the targeted gene
xuaA 637R	GTCTTAGGTGCCTGTAGGT	
ErmRamR	ACGCGTGCGACTCATAGAATTATTTCCTCCCG	
1249-E-RT-up_	TACGGCACCTCCTCAATATG	Transcriptional intergenic links
qRT_*xuaS* _708_Rev	CCCATCACGATCCCGTTCTT	
qPCR_*xuaS* _431_dir	CGGCAGGATTAAAGGGAGCA	
1251-S-RT-do	GTCCAAGCAGCAGAATTACAG	
1250-E-RT-up_	GAGACTTATATTGCAGCCCATC	
1252-S-RT-do	CTGCAAGGTCATCGTCTATTC	
qPCR_*xuaR* _277_dir	AAAGCAATTCAGCACCAGGG	
qRT-*xuaA* -525-rev	ACCGGTTCCTTCTTCTGTCAT	
qPCR_*xuaA* _2nd_378_dir	ATCTGGAGAACTCCCTGATGT	
qPCR_*xuaB* _584_rev	GCTGCTTCGTATAAACTGGGG	
1252-E-RT-up-2nd	AGGCGACACTTCACAATG	
1254-S-RT-do-3rd	CCCAGTGCGATTCTTTCAAC	
1253-E-RT-up_	AGCTCGTATAGATGGAGCTAAC	
1255-S-RT-do	GCCGCTTCCAACATTACC	
1254-E-RT-up_	GCCACTGGACTACACTCTG	
1256-S-RT-do	CCATCCGCTAAATGCTTACAG	
1255-E-RT-up-2nd	GAGTTGGTGGTACGTCATTC	
1257-S-RT-do	CTGAGCAATTCCCTGATTCTG	
1256-E-RT-up_	GCGGATGGAATCACTTGTATG	
1258-S-RT-do	GCCATAGCGAAATACTCTTCTC	
1257-E-RT-up_	GACCGCCAGGAATACATTAAC	
1259-S-RT-do	AATTTGGCTCGTTCCAGAC	
1258-E-RT-up_	CCGCGTTGCAATGAATATAGG	
1260-S-RT-do	TGCAATTATACGGCCGAATC	
1259-E-RT-up_	ACAATAACCGCTTGGAACC	
1261-S-RT-do	TGCCCACCGAGATATAGC	
1260-E-RT-up_	ACCCTGAGGCACTAATCC	
1262-S-RT-do	GAGTGCACGATTTCATCAGG	

Restriction sites are underlined (NdeI CATATG; NcoI CCATGG, XhoI CTCGAG, BamHI GGATCC, EheI/NarI GGCGCC), ATG is in boldface type
